# Dynamic Prediction of Survival in Cystic Fibrosis: A Landmarking Analysis Using UK Patient Registry Data

**DOI:** 10.1097/EDE.0000000000000920

**Published:** 2018-11-30

**Authors:** Ruth H. Keogh, Shaun R. Seaman, Jessica K. Barrett, David Taylor-Robinson, Rhonda Szczesniak

**Affiliations:** From the a Department of Medical Statistics, London School of Hygiene and Tropical Medicine, London, United Kingdom; b Medical Research Council (MRC) Biostatistics Unit, University of Cambridge, Cambridge, United Kingdom; c Department of Public Health and Policy, Farr Institute, HERC, University of Liverpool, Liverpool, United Kingdom; d Division of Biostatistics and Epidemiology and Division of Pulmonary Medicine, Cincinnati Children’s Hospital Medical Center, University of Cincinnati, Cincinnati, OH; e Department of Paediatrics, University of Cincinnati, Cincinnati, OH.

**Keywords:** Cox regression, Cystic fibrosis, Dynamic prediction, Landmarking, Longitudinal data, Patient registry, Personalized prediction, Survival

## Abstract

**Background:**

Cystic fibrosis (CF) is an inherited, chronic, progressive condition affecting around 10,000 individuals in the United Kingdom and over 70,000 worldwide. Survival in CF has improved considerably over recent decades, and it is important to provide up-to-date information on patient prognosis.

**Methods:**

The UK Cystic Fibrosis Registry is a secure centralized database, which collects annual data on almost all CF patients in the United Kingdom. Data from 43,592 annual records from 2005 to 2015 on 6181 individuals were used to develop a dynamic survival prediction model that provides personalized estimates of survival probabilities given a patient’s current health status using 16 predictors. We developed the model using the landmarking approach, giving predicted survival curves up to 10 years from 18 to 50 years of age. We compared several models using cross-validation.

**Results:**

The final model has good discrimination (C-indexes: 0.873, 0.843, and 0.804 for 2-, 5-, and 10-year survival prediction) and low prediction error (Brier scores: 0.036, 0.076, and 0.133). It identifies individuals at low and high risk of short- and long-term mortality based on their current status. For patients 20 years of age during 2013–2015, for example, over 80% had a greater than 95% probability of 2-year survival and 40% were predicted to survive 10 years or more.

**Conclusions:**

Dynamic personalized prediction models can guide treatment decisions and provide personalized information for patients. Our application illustrates the utility of the landmarking approach for making the best use of longitudinal and survival data and shows how models can be defined and compared in terms of predictive performance.

## Abstract 2

Supplemental Digital Content is available in the text.

Cystic fibrosis (CF) is an inherited, chronic, progressive condition affecting around 10,000 individuals in the United Kingdom and over 70,000 worldwide.^1,2^ In the United Kingdom, CF affects about 1 in 2500 live births.^3^ Children with CF are generally diagnosed in the first few months of life, with universal newborn screening implemented in 2007 in the United Kingdom, though some people with milder phenotypes are diagnosed in adulthood.^4^

Survival in CF has improved considerably over recent decades. Of individuals born around 1970, over half died before reaching their mid-teens to late teens.^5,6^ By contrast, the estimated median survival age for a person born with CF today in the United Kingdom is 48 for males and 44 for females.^1,7^ It is important to be able to provide patients with up-to-date information on their prognosis and to provide clinicians with information to guide treatment decisions, including listing for lung transplantation.

Data from national CF patient registries with longitudinal measures of health status and long-term follow-up have created the opportunity to develop models for predicting survival based on individual characteristics.^8,9^ Although there have been many studies of factors associated with survival in CF (Buzzetti et al^10^ and MacNeill^3^ for overviews), fewer have focused on prediction. We identified three models for survival prediction in UK patients, but all are based on small samples or subsets of patients.^11–13^ Survival prediction models in CF have been developed using national patient registries by Liou et al^14^ and Mayer-Hamblett et al^15^ (United States), Aaron et al^16^ (Canada), and Nkam et al^17^ (France). Until recently there have been (to our knowledge) no detailed studies of survival using the UK CF Registry. Keogh et al^18^ provided estimates of survival using UK CF Registry data given the baseline characteristics of sex, genotype, and age of diagnosis. In this article, we develop a model for personalized prediction of survival in the United Kingdom making use of time-dependent measures of health status.

The aims of this article are two-fold. Our first aim was to use data from the UK CF Registry to develop a dynamic survival prediction model that provides estimates of the probability of short-term, mid-term, and long-term survival given a patient’s current and past health status.^19^ We used the landmarking approach applied to UK CF Registry data on adults from 2005 to 2015,^20,21^ giving predicted survival curves up to 10 years from each landmark age, which can be any age post-diagnosis from 18 to 50. The model therefore provides predictions for individuals living with the CF who already survived to a given age. The model is dynamic in that it enables predictions to be updated over time, using updated measures of time-dependent predictors alongside a patient’s current age. Our second aim was to provide an example for other researchers of how to develop a dynamic prediction model using landmarking, illustrating the utility of this approach for making the best use of longitudinal and survival data, and showing how different models can be defined and compared in terms of their predictive performance.

## METHODS

### Design and Data Source

We undertook a landmarking analysis using data from the UK CF Registry, a national, secure database sponsored and managed by the Cystic Fibrosis Trust.^19^ The Registry was established in 1995 and records demographic data and longitudinal health data on nearly all people with CF in the United Kingdom, to date capturing data on over 12,000 individuals. National Health Service (NHS) Research Ethics approval has been granted for the collection of data into the Registry. Each patient or their parent provided written informed consent for collection of data in the Registry and use of pseudonymized data in research. In the United Kingdom, CF patients are treated in specialist centers and data for the Registry are collected in a standardized way at designated (approximately) annual visits. Data collected cover over 250 variables in several domains, alongside mortality data. We restricted our analyses to a set of 17 variables (Table 1) recorded routinely in the Registry and previously found to be associated with survival, based on a review of the literature.^3,10,11,13,15–17,22–28^ This set consists of three baseline variables—sex, genotype (F508del alleles), and age of diagnosis—as well as calendar year, and 13 internal time-dependent variables: forced expiratory volume in 1 second as percentage predicted (FEV1%), forced ventricular capacity as percentage predicted (FVC%), height, weight, infection status for four organisms (*Pseudomonas aeruginosa*, *Staphylococcus aureus*, *Burkholderia cepacia*, Methicillin-resistant *Staphylococcus aureus* [MRSA]), CF-related diabetes, pancreatic insufficiency, days in hospital on intravenous (IV) antibiotics, days at home on IV antibiotics, and other hospitalization. We calculated FEV1% and FVC% using the Global Lung Initiative (GLI) equations.^29^ We investigated using body mass index (BMI) instead of weight and height but found that models including weight and height separately were better fitting, based on Akaike’s Information Criterion.^30^ The two variables for days on IV antibiotics are used as surrogate indicators for pulmonary exacerbations.^31,32^

**Table 1.**
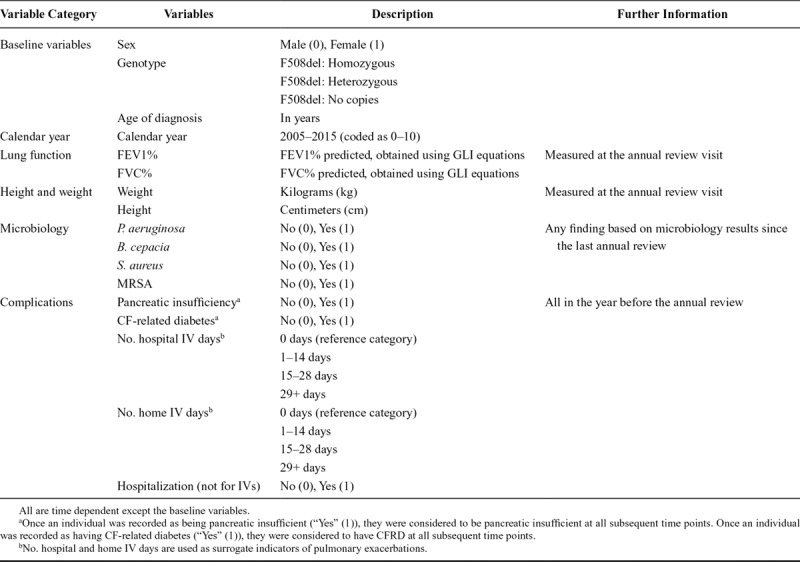
Variables Considered as Predictors

Analyses are based on follow-up during the study period 2005–2015, so that some individuals have at least 10 years of follow-up, enabling estimation of survival up to 10 years. We therefore excluded individuals who died or were lost to follow-up before 2005. In order to focus on adults, we only used data on individuals from 18 years of age onward during the study period.

### Landmarking Approach

The landmarking approach for dynamic prediction of survival was first described by van Houwelingen.^20^ A detailed account is provided by van Houwelingen and Putter.^21^ In brief, at a given age (a “landmark age”) from which a prediction is to be made, the data are restricted to individuals who have not yet had the event (in this case, death) or been censored. Values of predictor variables available up to the landmark age are used as covariates in a model for the probability of survival up to some time horizon, conditional on survival to the landmark age. Typically, the focus is on survival to a single time horizon (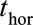), e.g., 2 years after the landmark age (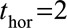), and censoring is imposed at 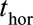 so that only events up to that time are used in the survival analysis. For a chronic condition like CF, however, it is of interest to study survival to several time horizons. We use the Cox model and its extensions to model survivor curves up to 10 years after each landmark age.

Landmark data sets were created from landmark ages 
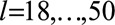
 (eFigure 1; http://links.lww.com/EDE/B407, eTable 1; http://links.lww.com/EDE/B407, eAppendix 1; http://links.lww.com/EDE/B407). Data on individuals over 50 years of age are sparse. The 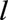
th landmark data set included all individuals known to be alive at age 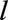 during 2005–2015, who had not received a transplant before age 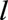, who were diagnosed with CF before age 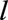, and who joined the Registry before age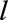. Individuals lost to follow-up before age 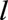 were excluded. We excluded people who received a transplant before age 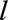 because the variables of importance for survival in transplanted patients are likely to be quite different from those of importance for untransplanted individuals.^33^ Individuals transplanted after age 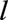 were included in the 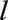
th landmark data set, and their deaths were counted as events in the survival analysis. The predictors in the 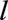
th landmark data set were the three baseline variables, calendar year, and variables that summarize the measurements of the remaining 13 time-dependent predictors up to age 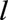. We summarize time-dependent measurements in two ways. First, we used the most recently available measure at time 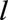 of each time-dependent variable. This “last-observation-carried-forward” approach was used in the original descriptions of landmarking.^20,21^ Second, we fitted a mixed effects model to data available on time-dependent variables up to the landmark age and used the resulting fitted values and slopes at the landmark age as predictors because some studies have suggested that this makes better use of the data than last-observation-carried-forward approach.^34–36^ We implemented this two-stage landmarking approach by fitting a multivariate mixed model to three continuous time-dependent variables—FEV1%, FVC%, and weight—up to each landmark age (eAppendix 2; http://links.lww.com/EDE/B407, eTable 2; http://links.lww.com/EDE/B407).

We created a single stacked data set by stacking the 33 landmark data sets (
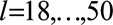
), for use in pooled models (see below). Many individuals appear multiple times in the stacked data set because they are eligible for several landmark data sets. Robust standard errors were used to account for this.

### Model Building

The aim was to obtain a dynamic prediction model that performs well for predicting 2-, 5-, and 10-year survival from each landmark age. We considered a number of multivariable Cox models (Table 2) before selecting a final model based on assessment of their predictive performance. Further details on the models and on how predicted survival probabilities were obtained are given in eAppendix 2; http://links.lww.com/EDE/B407.

**Table 2.**
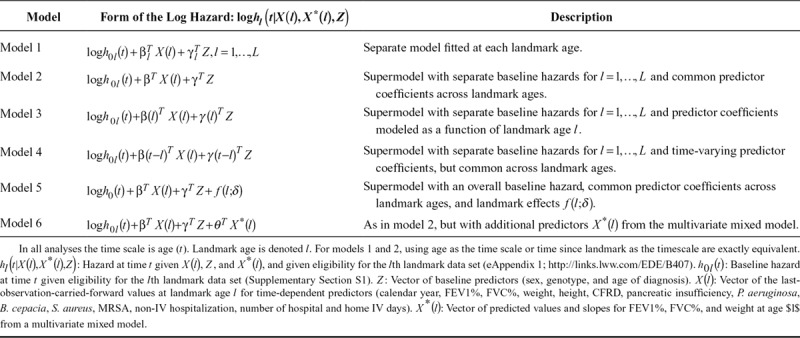
Summary of Dynamic Prediction Models Investigated

Models 1–5 use the last-observation-carried-forward values for the 13 time-dependent predictors. We began by fitting separate survival models from each landmark age 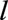 (model 1). An alternative is to fit a pooled model (a “supermodel”) to the stacked data set. The simplest supermodel (model 2) allowed a separate baseline hazard for each landmark age, but assumed common predictor coefficients across all landmark ages. Models 1 and 2 were initially fitted using a time horizon of 10 years (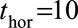), which enables us to obtain predicted survival probabilities for any time up to 10 years after the landmark age. We also investigated whether 2- and 5-year survival could be better predicted by using 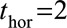 and 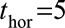, respectively. One might expect to better predict 2-year survival (for example) by using 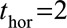 instead of 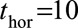 because the effects of time-dependent variables are expected to change less over 2 years than 10 years. However, this was not found to be the case and all subsequent models were fitted with 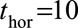. Because we found that the supermodel gave better predictive performance, subsequently investigated models were all extensions of model 2.

Model 3 allows predictor coefficients (log hazard ratios) to vary smoothly with 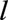. Model 4 allows the predictor coefficients to vary with time since landmark 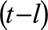. Model 5 uses a common baseline hazard with the impact of landmark age on the hazard modeled using regression terms. Model 6 extends model 2 by using the fitted value and slope at each landmark age for each of FEV1%, FVC%, and weight from the multivariate mixed models (one for each landmark age) as additional time-dependent predictors (as well as the last-observation-carried-forward values). By incorporating slopes from the mixed models, the prediction model includes information about trajectories of FEV1%, FVC%, and weight up to each landmark age. For height and the categorical time -dependent variables, we used last-observation-carried-forward approach in all models. In all models continuous variables were assumed to have linear effects; modeling them using splines brought negligible changes in predictive performance.

### Model Assessment

We divided the data into a training-plus-validation set—an 80% random sample of the stacked data, stratified by landmark age—and a “holdout” set—the remaining 20%.^37^ The training-plus-validation set was used for model development and assessment. Details are given in eAppendix 3; http://links.lww.com/EDE/B407.

We compared the predictive performances of different models in terms of discrimination, using the C-index,^38–40^ and prediction error, using the Brier score.^41,42^ C-indexes and Brier scores were calculated separately for each landmark age for prediction of 2-, 5-, and 10-year survival. We also obtained overall C-indexes and Brier scores across landmark ages for 2-, 5- and 10-year survival. A Monte–Carlo cross-validation procedure was used to avoid overoptimism about predictive performance.^43^

We selected the model with the best predictive performance as the final model, though where several models had similar performance we favored a simpler model. The final model was applied to the holdout data to estimate its performance in a new set of individuals. Last, the final model was fitted to the complete data and is reported in full for use by other researchers.

We performed all analyses using R. eAppendix 4; http://links.lww.com/EDE/B407 provides details on software.

## RESULTS

### Data Overview

**Figure 1.**
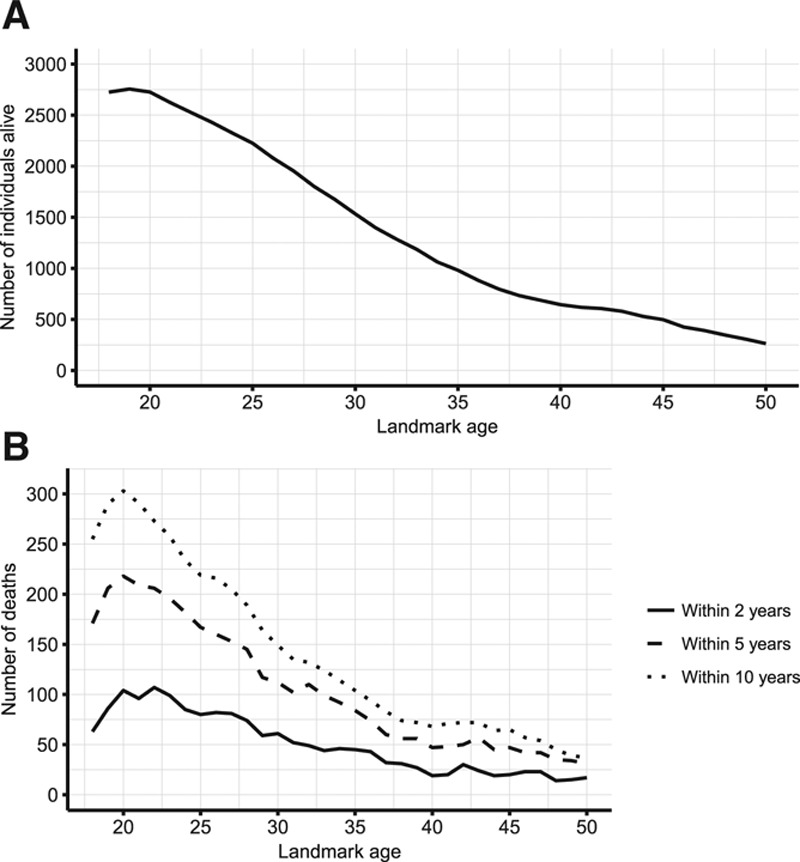
Overview of number of individuals in each landmark data set. A, Number of individuals alive at each landmark age at any point during the study period. B, Number of deaths within 2, 5, and 10 years after each landmark age, among those alive at each landmark age.

The stacked data set has 43,592 rows and 6181 unique individuals, of whom 931 died within 10 years of follow-up (eAppendix 2; http://links.lww.com/EDE/B407). Censoring is owing to the end of follow-up at the end of 2015, rather than loss to follow-up (eAppendix 2; http://links.lww.com/EDE/B407). Many individuals appear in multiple landmark data sets. eFigure 1; http://links.lww.com/EDE/B407 illustrates how the data arose. Figure 1 summarizes the number of individuals in each landmark data set and the number of deaths within 2, 5, and 10 years of each landmark age. eTable 1; http://links.lww.com/EDE/B407 gives more detailed information. eTable 3; http://links.lww.com/EDE/B407 summarizes the predictors at landmark ages 20, 30, 40, and 50.

### Comparison of Dynamic Prediction Models

Overall C-indexes and Brier scores from models 1 to 6 are shown in Table 3. Model 1, in which separate models were fitted from each landmark, gave overall C-indexes of 0.841 for 2-year survival, 0.811 for 5-year survival, and 0.771 for 10-year survival, and corresponding Brier scores of 0.038 for 2-year survival, 0.082 for 5-year survival, and 0.147 for 10-year survival, indicating better predictive performance for short-term survival. A supermodel fitted across landmark ages (model 2) brought gains in terms of both discrimination (C-indexes) and prediction error (Brier scores). The C-indexes increased to 0.873 for 2-year survival, 0.843 for 5-year survival, and 0.804 for 10-year survival, and the Brier scores reduced to 0.036 for 2-year survival, 0.076 for 5-year survival, and 0.133 for 10-year survival. Landmark age-specific C-indexes and Brier scores (eFigures 2 and 3; http://links.lww.com/EDE/B407) show that the gains in predictive performance from using the supermodel are particularly important for older landmark ages. This is because there are fewer data at those ages and hence more to be gained by drawing strength from other landmark ages by using a supermodel.

**Table 3.**
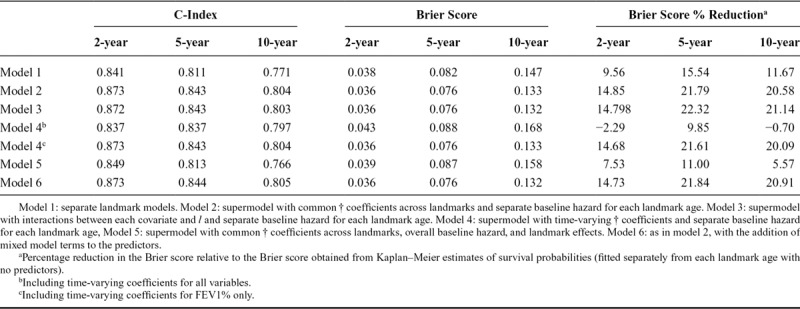
Overall C-Indexes, Brier Scores, and Brier Score Percentage Reductionsa for Prediction of 2-, 5-, and 10-Year Survival From Models 1–6

Allowing the predictor coefficients to depend on landmark age in a smooth way (model 3) resulted in very similar results to model 2. Including time-varying coefficients for all predictors (model 4) resulted in worse predictive performance compared with model 2. Restricting the time-varying coefficients to FEV1%, the strongest predictor, gave very similar results to model 2. Using splines instead of a linear form for the time-varying coefficients did not bring any improvements. This lack of advantage of using time-varying coefficients in part reflects our finding that using a shorter time horizon (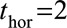 or 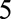) did not improve prediction. Using a common baseline hazard, with the impact of landmark age modeled using regression terms (model 5), resulted in considerably worse predictive performance than model 2.

Inclusion of the fitted values and slopes from mixed models for FEV1%, FVC%, and weight in addition to the last-observation-carried-forward terms brought small improvements in the C-indexes and Brier scores. Further investigations found that including the mixed model terms without the corresponding last-observation-carried-forward terms resulted in worse predictive performance than models 2 and 6.

### Final Model

Based on the above comparisons, we selected model 2 as the final model: increasing model complexity had not resulted in improvements in predictive performance, suggesting a trade-off between increased complexity and estimation of more parameters. While there were small gains in predictive performance from using mixed models for three of the continuous variables (model 6), these were fairly negligible and came at the expense of a substantially more complicated procedure for obtaining predicted survival probabilities. Also, model 2 requires only the most recent values of predictors at the landmark age, while the mixed modeling approach (model 6) requires a series of measures up to the landmark age. Furthermore, model 2 is more straightforward to explain and report to potential users.

eFigure 4; http://links.lww.com/EDE/B407 shows calibration plots for the final model for landmark ages 20, 30, 40, and 50, which compare model-based predicted survival probabilities with “observed” probabilities. For 2-year and 5-year survival, the points lie close to the line of equality, indicating good agreement between predicted probabilities from the model and the observed probabilities. There is also good agreement for 10-year survival for landmark ages 20, 30, and 40. At landmark age 50, the agreement between predicted and observed 10-year survival probabilities is less good, which may be partly owing to sparse data at the older ages. These results indicate that the model is well calibrated for prediction of 2- and 5-year survival from all landmark ages, and for 10-year survival at least up to age 40.

### Application in the Holdout Data

The final model was fitted to the complete training-plus-validation data and applied to the holdout data to demonstrate its use in practice. The resulting overall C-indexes were for 0.854 for 2-year survival, 0.843 for 5-year survival, and 0.815 for 10-year survival. The corresponding overall Brier scores were 0.034, 0.077, and 0.125, representing percentage reductions in prediction error against the Kaplan–Meier estimates of survival probabilities of 12.22%, 20.92%, and 23.86%. eTable 4; http://links.lww.com/EDE/B407 summarizes observed survival within groups defined by the predicted survival probabilities.

### Full Model Specification

**Figure 2.**
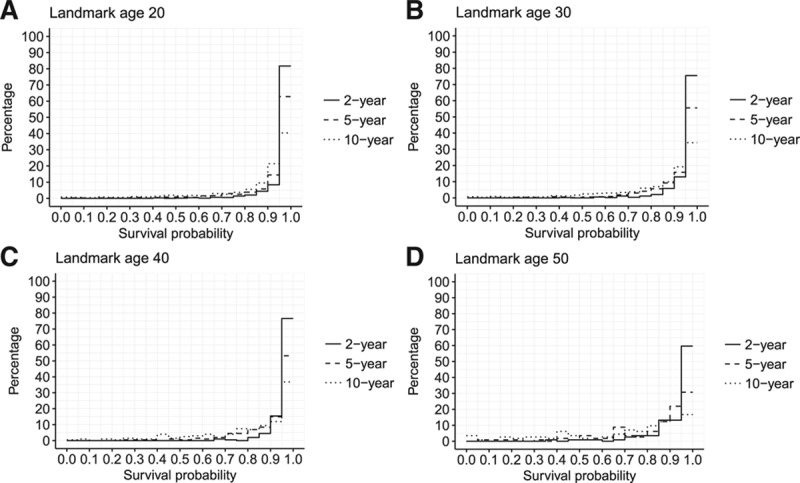
Plots showing the distribution of 2-, 5-, and 10-year survival probabilities from landmark ages (A) 20, (B) 30, (C) 40, and (D) 50 for individuals in the Registry at those ages between 2013 and 2015. [This plot is shown in color in eFigure 9; http://links.lww.com/EDE/B407.]

We fitted the final model to the complete data (the training-plus-validation and holdout data combined). Estimated baseline hazards 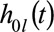 are given in at a web link given in eAppendix 5; http://links.lww.com/EDE/B407; in combination with the regression coefficients in Table 4, these provide a full specification of the dynamic prediction model. Higher FEV1%, FVC%, and weight were strongly associated with reduced hazard. *B. cepacia* infection, CF-related diabetes, and more hospital days on IV antibiotics were strongly associated with increased hazard. Using the final model fitted to the complete data, we calculated 2-, 5-, and 10-year predicted survival probabilities from 20, 30, 40, and 50 years of age for individuals in the CF Registry at these ages during the most recent 3-year period for which data were available (2013–2015). eFigures 5–8; http://links.lww.com/EDE/B407 illustrate typical profiles of individuals within groups defined by predicted survival probabilities and show corresponding predicted survivor curves, illustrating in particular how FEV1%, FVC%, weight, CF-related diabetes (CFRD), and IV days are associated with survival, Figure 2 shows the distributions of the predicted probabilities. At 20 years of age, over 80% of individuals had a greater than 95% probability of 2-year survival and over 35% of 10-year survival. At landmark ages 30, 40, and 50, over 75% of individuals had a greater than 90% probability to survive 2 years, and over 50% had a greater than 90% probability to survive 5 years. These plots further demonstrate how the model could be used to identify patients at greatest risk and those with a good prognosis.

**Table 4.**
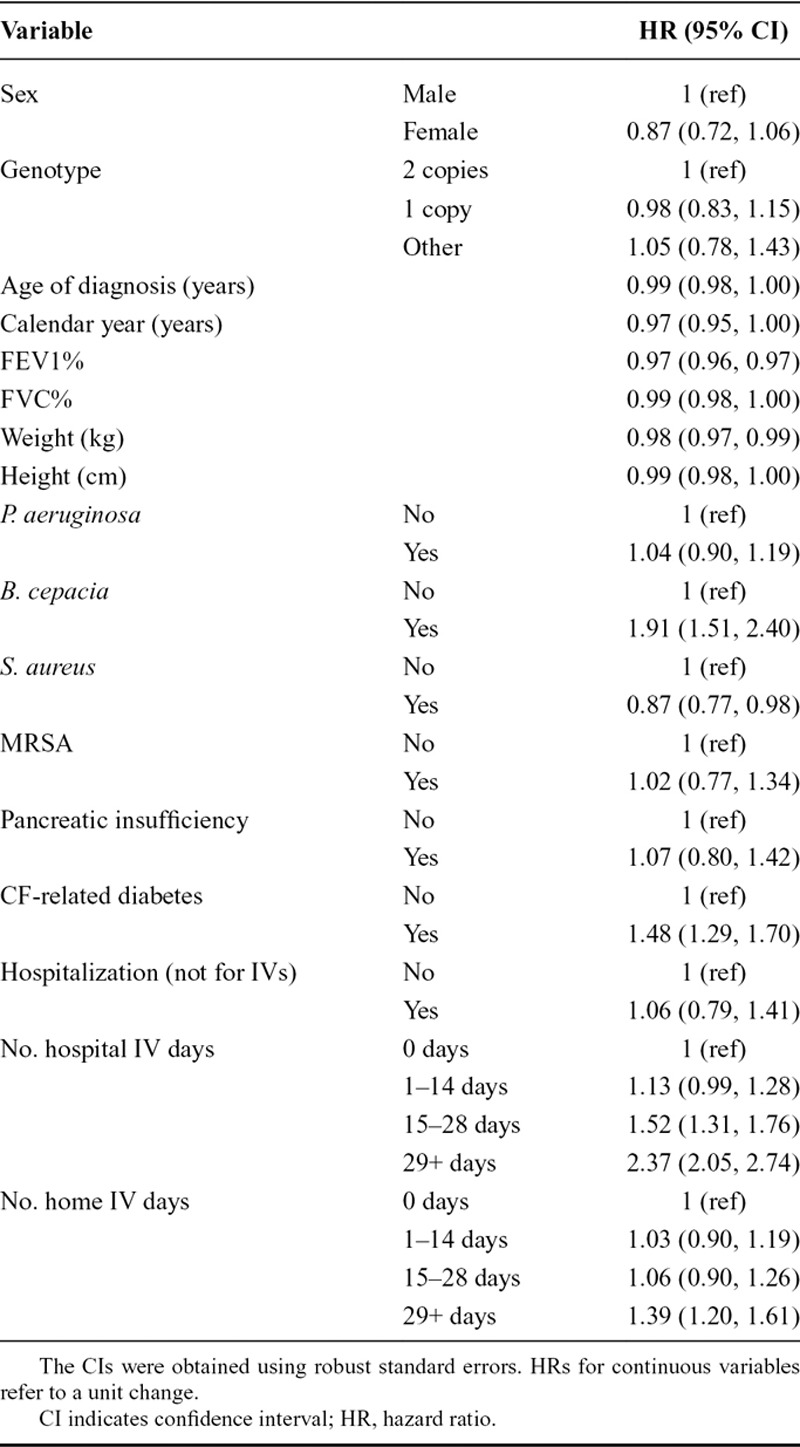
Results From Fitting the Final Selected Model to the Complete Data

## DISCUSSION

We have developed a model for dynamic prediction of survival for people with CF in the United Kingdom using UK CF Registry data. We used a landmarking approach applied to CF data to our knowledge for the first time, making efficient use of the longitudinal data, by using information from the same individual at several ages and incorporating updated measures of health status. The model enables predictions of survival up to 10 years for adults with CF up to 50 years of age and can be used to identify high-risk patients, making use of information on 16 variables. R code for obtaining estimated survival probabilities from the final model is provided at https://github.com/ruthkeogh/landmark_CF. There are several potential roles for practical use of the model, including for guiding treatment decisions, informing referral for lung transplantation,^44^ and providing personalized information going far beyond the population-level statistics that are currently available, which is important for patients.

We have outlined a systematic approach to development of a dynamic prediction model using landmarking, incorporating the assessment of models of different levels of complexity by comparing their predictive performance. There have been relatively few practical applications of landmarking.^34,45,46^ Unlike previous applications, we have provided predicted survival curves instead of focusing on a single time horizon, and we provided results on model performance for 2-, 5-, and 10-year survival. Prediction of long-term survival is of particular relevance for chronic conditions such as CF, and ours is to our knowledge the first prediction model based on UK CF Registry data. Of the three earlier prediction models using national patient registry data, two used logistic regression,^14,17^ and so did not handle censoring, and did not make efficient use of the longitudinal data. Aaron et al^16^ used a stochastic process model. No previous prediction models in CF have considered survival to more than one time point or beyond 5 years.^12–17,22,25^ Comparisons of predictive performance with models obtained in other populations are summarized in eAppendix 6; http://links.lww.com/EDE/B407. Future work may result in new models for the UK population that could be compared with ours, and it is important that similar measures of predictive performance are presented across studies to facilitate comparisons. We used the landmarking approach to perform dynamic prediction. An alternative approach uses joint modeling of the longitudinal and survival processes.^47–49^ Landmarking had several strengths over joint modeling for this application. First, landmarking enabled us to handle transplanted individuals in a straightforward way. We excluded previously transplanted individuals at each landmark age but retained post-transplant deaths in the data set for estimating survival after each landmark age. Our predictions therefore refer to individuals who are untransplanted at the time of making the prediction. Development of a prediction model for post-transplant survival is an area for further work. It is not clear how transplanted individuals should be handled in the joint modeling approach, especially using readily available software. Second, the set of predictors included 12 endogenous time-dependent variables of different types (continuous, categorical, binary). Although joint modeling has recently been extended for use with multivariate longitudinal outcomes,^50^ its feasibility for use with a large number of such variables of different types remains in question. The two-stage landmarking approach,^34–36^ which used mixed models for continuous time-dependent predictors (model 6), did not result in material gains compared with using the last-observation-carried-forward method. Landmarking also has the advantage of being based on methods, notably Cox regression, that are familiar to a clinical audience, which facilitates its explanation. Recent comparisons of landmarking with joint modeling using simulation studies have tended to find joint modeling to perform slightly better than landmarking.^35,36,51^ However, they have focused on simple simulation scenarios favoring the joint model.

A major strength of our study is the use of the UK CF Registry data to create the dynamic prediction model. The Registry collects longitudinal data on almost all UK CF patients, and the structured data collection means that there are little missing data and little loss to follow-up. A limitation is that predicted survival probabilities cannot account for improvements in survival that are not yet known about, e.g., owing to new treatments.^52,53^ However, treatments manifest themselves in measures of health status, and so it is likely that the prediction model could still apply. That is, the distribution of health status measures in the CF population may change, but the associations of health status measures with survival remain the same. The standardized format of the Registry data collection means that the model could be assessed and updated if necessary after a few years.

We selected a set of predictors previously associated with survival in CF and collected routinely in the Registry.^3,10^ FEV1% is the strongest predictor, though predictive performance is improved by incorporating the additional variables (eTable 5; http://links.lww.com/EDE/B407). Further investigations using variable selection techniques tended to result in a model containing most of the variables. Extensions of variable selection techniques to the context of dynamic prediction remain an area for further methodologic work. There are many other variables in the Registry, and an area for further work is to investigate whether using additional variables could improve predictive performance. We took the decision not to use data on treatment use as predictors. As noted above, the impact of treatments on survival is expected to manifest primarily via the health status measures used as predictors. Further investigations also found that adding information on use of two treatments did not materially improve prediction (eTable 5; http://links.lww.com/EDE/B407). Furthermore, the models created in this work are designed with prediction in mind, and the estimated coefficients associated with the predictor variables do not necessarily represent causal effects. Inclusion of treatment variables could create danger of misinterpretation of the impacts of treatment on survival prediction curves as causal effects, which could result in inappropriate withholding of treatment if treatment is (noncausally) associated with worse prognosis. Estimation of treatment effects using patient registry data is an area of growing interest^54,55^ but involves a separate question from that focused on in this article.

Our model is for adults with CF. There are relatively few deaths in CF patients under 18 years of age in the United Kingdom, and different variables may be important for survival prediction in children.^12,56^ We restricted to predictions for adults up to 50 years of age because the data above 50 years of age are sparse. Investigations into the health of older people with CF are of interest.

In summary, we have developed a novel landmarking model for dynamic prediction of survival for people with CF in the United Kingdom. Further work involves the practical implementation of our model in a form suitable for use by clinicians, potentially as an add-on to patient information that can already be viewed via the Registry interface. In addition, it is important that patients and caregivers are supported to interpret personalized survival predictions.^57–59^

## ACKNOWLEDGMENTS

We thank people with cystic fibrosis and their families for consenting to their data being held in the UK Cystic Fibrosis (CF) Registry and NHS teams in CF centers and clinics for the input of data into the Registry. We also thank the UK Cystic Fibrosis Trust and the Registry Steering Committee for access to anonymized UK CF Registry data. The analyses presented in this paper use a data set resulting from a cleaning process undertaken by the CF Epidemiological Network, which was funded by a Strategic Research Centre Grant from the Cystic Fibrosis Trust.

## Supplementary Material
