# Supplementary material for: Dynamic Prediction of Survival in Cystic Fibrosis: A Landmarking Analysis Using UK Patient Registry Data
Source: Epidemiology. 2018 Nov 30;30(1):29–37. doi: 10.1097/EDE.0000000000000920 (PMC6276867; doi:10.1097/EDE.0000000000000920)
Supplement: Supplementary file 1 [file ede-30-029-s001.pdf]

Supplementary materials for:

Dynamic prediction of survival in cystic fibrosis: A landmarking analysis using UK patient registry data

eAppendix 1. Creation of landmark data sets

eFigure 1 illustrates how the landmark data sets arose. An individual was included in the landmark data set at age l if they met *all* of the following criteria:

- They reached age l between 1st January 2005 and 31st December 2015.
- They joined the Registry prior to reaching age l. The date of joining the Registry is the date of the first annual review at which data were obtained.
- They were diagnosed with CF prior to reaching age l.
- They have not received an organ transplant of any type prior to reaching age l.
- They have measures of all time-dependent variables recorded prior to reaching age l.

We refer to an individual as "eligible for the lth landmark data set" if she/he satisfied these five conditions. eTable 1 summarises the landmark data sets in terms of number of individuals, number of deaths within 2, 5 and 10 years of the landmark age, and number of censorings.

eAppendix 2. Survival prediction models

Time scale and follow-up

In all models the time origin is date of birth and analyses are performed using left-truncation at the landmark age. The censoring time was the earliest of death, 31st December 2015 and a specified time horizon t_{hor}. Since dates of birth and death were only available in month/year format, the day was imputed as the 15th of the month. For example, an individual aged 18 on 1st January 2005 (who has been diagnosed, joined the Registry, and not received a transplant) contributes up to 11 years of follow-up until the end of 2015 to the landmark data set for age 18 and up to 10 years of follow-up for the landmark dataset for age 19 (if they do not die or have a transplant between ages 18 and 19), and so on. An individual aged 18 on 1st January 2014 contributes up to 2 years of follow-up to the landmark data set for age 18 and up to 1 year of follow-up for the landmark dataset for age 19.

The UK CF Registry aims to capture deaths from all causes. Of the 931 deaths used in this study, 775 (83.2%) were due to respiratory or cardiorespiratory failure, 55 (5.9%) were transplantation-related, 13 (1.4%) were due to liver disease or failure, 9 (1.0%) were due to cancer, 9 (1.0%) were due to trauma or suicide, 34 (3.7%) were due to "other causes" (recorded in a separate field and including "End state cystic fibrosis" and "Haemoptysis"), 35 (3.9%) were due to an unknown cause, and for 1 individual the cause was not recorded.

We assumed that all deaths are captured and the main results presented assume censoring is entirely administrative. In a sensitivity analysis we treated individuals not recorded at an annual follow-up for over 2 years as lost-to-follow-up. This did not materially alter the results – the C-indexes for 2-5- and 10-year survival from the final model (Model 2) were 0.874, 0.847, 0.807 respectively, and corresponding Brier scores were 0.036, 0.075, 0.130.

Landmark survival models

We let Z denote the vector of baseline predictors (sex, genotype and age of diagnosis) and $X(l)$ denote the vector of the last-observation-carried-forward (LOCF) values for time-dependent predictors (calendar year, FEV%, FEV%, weight, height, CFRD. pancreatic insufficiency, *Pseudomonas aeruginosa*, *Burkholderia cepacia*, *Staphylococcus aureus*, Methicillin-resistant *Staphylococcus aureus* (MRSA), non-IV hospitalization, number of IV days) at landmark age l.

Model 1 for the log conditional hazard is

$$\log h_l(t|X(l),Z) = \log h_{0l}(t) + \beta_l^T X(l) + \gamma_l^T Z, l = 1, \dots, L \qquad \text{Model 1}$$

where $h_{0l}(t)$ is the baseline hazard at age t conditional on eligibility for the lth landmark data set, and β_l and γ_l are vectors of log hazard ratios specific to landmark age l. Model 1 is in fact L models, which are fitted in each landmark data set $l = 1, \dots, L$.

Model 2 for the log conditional hazard is

$$\log h_l(t|X(l),Z) = \log h_{0l}(t) + \beta^T X(l) + \gamma^T Z \qquad \text{Model 2}$$

where $h_{0l}(t)$ is again the baseline hazard at age t conditional on eligibility for the lth landmark data set ($l = 1, \dots, L$). β and γ are vectors of log hazard ratios, which are assumed to be the same for all l. Model 2 therefore allows a separate baseline hazard from each landmark age, but common predictor coefficients across all landmark ages. It is fitted in the stacked data set using Cox regression with a stratified baseline hazard.[1,2] We note that for Models 1 and 2, using age as the time scale or time-since-landmark as the timescale are exactly equivalent.

Models 1 and 2 make the proportional hazards assumption that the association of the predictors $X(l)$ and Z with the hazard is the same over time since l, i.e. that the β_l and β parameters are not time-dependent. Models 1 and 2 were initially fitted using a time horizon of 10 years ($t_{hor} = 10$), which enables us to obtain predicted survival probabilities for any time up to 10 years. We also investigated whether 2-year and 5-year survival could be better predicted by using a shorter time horizon by fitting Models 1 and 2 using $t_{hor} = 2$ and $t_{hor} = 5$ respectively.

Model 3 extends Model 2 by allowing the log hazard ratios to depend on l in a smooth way:

$$\log h_l(t|X(l),Z) = \log h_{0l}(t) + \beta(l)^T X(l) + \gamma(l)^T Z \qquad \text{Model 3}$$

where $\beta(l)$ and $\gamma(l)$ denote vectors of log hazard ratios that are functions of l. We considered linear forms $\beta(l) = \beta_0 + \beta \times (l - 18)$ and $\gamma(l) = \gamma_0 + \gamma \times (l - 18)$ and restricted cubic spline forms with knots at 18, 30, 40 and 50. The results reported in Table 3 of the main text are from the analysis using the linear form for $\beta(l)$, as using restricted cubic splines did not materially improve predictive performance.

In Model 4 the supermodel was extended to allow time-varying coefficients, with the association between the predictors and the hazard dependent on time-since landmark $(t - l)$:

$$\log h_l(t|X(l),Z) = \log h_{0l}(t) + \beta(t-l)^T X(l) + \gamma(t-l)^T Z \qquad \text{Model 4}$$

where $\beta(t-l)$ and $\gamma(t-l)$ denote vectors of log hazard ratios that are functions of $t - l$. We considered linear forms $\beta(t-l) = \beta_0 + \beta \times (t - l)$ and $\gamma(t-l) = \gamma_0 + \gamma \times (t - l)$ and restricted cubic spline forms with knots at $t - l = 2,5,8$. The results reported in Table 3 of the main text are from the analysis using the linear form for $\beta(t-l)$, as using restricted cubic splines did not materially improve predictive performance.

Model 5 uses an overall baseline hazard instead of separate baseline hazards for each landmark age, with the impact of landmark age modelled using regression terms:

$$\log h_l(t|X(l),Z) = \log h_0(t) + \beta^T X(l) + \gamma^T Z + f(l;\delta) \qquad \text{Model 5}$$

where $h_0(t)$ is a common baseline hazard and $f(l;\delta)$ is a function of landmark age. We used a restricted cubic spline form for $f(l;\delta)$ with knots at 18, 30, 40 and 50.

In Model 6 we extended Model 2 by adding the fitted values and slopes from the multivariate mixed model (see below) for FEV%, FVC% and weight to the set of time-dependent predictors at each landmark age:

$$\log h_l(t|X(l),Z) = \log h_{0l}(t) + \beta^T X(l) + \gamma^T Z + \theta^T X^*(l) \qquad \text{Model 6}$$

where $X^*(l)$ denotes the vector of predicted values and slopes for FEV%, FVC% and weight from the multivariate mixed model.

All models were fitted by maximum partial likelihood.

Multivariate mixed model

A multivariate linear mixed model for FEV1%, FVC%, BMI and weight was fitted to the repeated measures up to landmark age l $(l = 1, \ldots, L)$ for individuals in the landmark data set at age l. Separate models were fitted for each landmark age. The longitudinal variables were modelled as a linear function of age with a random intercept and slope. We also included fixed effects of all the other predictors, including both baseline and time-dependent predictors. For each individual in landmark dataset l $(l = 1, \ldots, L)$ the individual fitted values and slopes for FEV1%, FVC% and weight at age l were obtained. The numbers of longitudinal measurements used in the multivariate mixed models are summarised in eTable 2.

Predicted survival probabilities

From each model the predicted survival probability to time t after the landmark age, conditional on survival to the landmark age, on baseline variables Z and on values of time-dependent predictors at the landmark age $X(l)$, $S(l+t|X(l),Z,T>l)$, was obtained using the relationship

$$S(l+t|X(l),Z,T>l) = \exp\left\{-\int_l^{l+t} h(u|X(l),Z,u>l)\,du\right\}$$

For models without time-varying hazard ratios (Models 1-3 and 5-6) we used the estimator:

$$\hat{S}(l+t|X(l),Z,T>l) = \exp\left\{-e^{\hat{\beta}^T X(l)+\hat{\gamma}^T Z}\sum_{l<u\le l+t}\hat{h}_{0u}\right\}$$

where $\hat{h}_{0u}$ denotes the baseline hazard at time u estimated from the increments in Breslow's estimate of the cumulative baseline hazard and the sum is over event times.[3] For Model 4, which has time-varying hazard ratios, we used the estimator

$$\hat{S}(l+t|X(l),Z,T>l) = \exp\left\{-\sum_{l<u\le l+t}\hat{h}_{0u}e^{\hat{\beta}^T(u-l)X(l)+\hat{\gamma}^T(u-1)Z}\right\}$$

eAppendix 3. Model assessment

Overview

Models were assessed and compared based on the "3-in-1" procedure described by Yong et al (2013), which incorporates model building using cross-validation, final model choice, and statistical inference.[4] The data were first divided into a "training+validation" (TV) set and a "holdout" set. The TV set is used in the model development and assessment. The holdout set is reserved for applying the selected model at the end. No models are fitted using the holdout data. The TV set is a sample of 80%

from the stacked data, stratified by landmark age. The holdout set is formed from the remaining 20% of individuals at each landmark age. Some individuals appear in both the TV and holdout stacked data sets, but not with the same landmark age.

For model assessment we used the C-index,[5–8] the Brier score,[9,10] and percentage reduction in the Brier score relative to the null model (i.e. the model excluding all predictors, using Kaplan-Meier estimates).[11] The C-index and Brier scores were obtained using inverse probability of censoring weights. For Model 4 we accommodated the time-varying coefficients into the estimation of the C-Index and Brier score.[8]

A Monte-Carlo cross-validation procedure was used within the TV data set to avoid over-optimism due to overfitting [12]. The procedure was as follows:

(i) An 80% stratified random sample, with stratification by landmark age l, was obtained from the TV data set.
(ii) The model was fitted on the 80% sample.
(iii) The fitted model was used to obtain predicted survival probabilities to a given time from each landmark age l (see below) for the 20% not in the sample.
(iv) Model performance measures (C-index, Brier score, and percentage reduction in the Brier score) were obtained in the 20% not in the sample on which the model was fitted.
(v) Steps (i)-(iv) were repeated 200 times and we obtained the average C-index, Brier score and Brier score reduction across the 200 samples.

Model assessment measures were obtained for 2-year, 5-year and 10-year survival from each landmark age. Therefore there are 99 averaged C-indices and Brier scores for each model (33×3, where 33 is the number of landmark ages 18-50). For each model we also obtained an overall C-index and Brier score which are not age-adjusted. Further details are given below. To simplify the notation we give the details of the C-index and Brier score as if applied to the complete stacked data (the TV and holdout data combined).

Truncated C-Index

The following description of the C-index follows that of Gerds et al..[7] Let T_i and C_i denote respectively the event time and censoring time for individual i. We observe $\tilde{T}_i = \min(T_i, C_i)$ and the event indicator $\Delta_i = 1(T_i < C_i)$. Let $\hat{S}_l(l+t|X(l),Z)$ denote the estimated probability of survival beyond age $l+t$ conditional on survival to age l and given predictor values $X(l),Z$ at age l. The truncated C-index is

$$C_l(t) = E_{ij}\{1\{\hat{S}_l(l+t|X_i(l),Z_i) < \hat{S}_l\big(l+t|X_j(l),Z_i\big)\}|T_i < T_j, T_i \leq l+t, T_i > l, T_j > l\}$$

where the expectation is with respect to two subjects i,j, both alive at age l ($T_i > l$). Not all pairs of individuals i,j are comparable. We can compare two individuals who both have the event prior to age $l+t$; two individuals, one of whom has the event prior to age $l+t$ and the other of which is known to be alive (censored) at age $l+t$. We cannot compare two individuals who are both known to be alive (censored) at age $l+t$, two individuals both censored before age $l+t$, or a pair in which one individual has the event and the other is censored before the other's event time. The fact that not all pairs of individuals can be compared is handled using inverse probability of censoring weights (IPCW). The truncated C-Index can be expressed as

$$C_l(t) = \frac{E_{ij}\{1\{\hat{S}_l(l+t|X_i(l),Z) < \hat{S}_l(l+t|X_j(l),Z_j)\}|T_i > l, T_j > l\}E_{ij}\{T_i < T_j, T_i \leq l+t|T_i > l, T_j > l, X_i(l), X_j(l)\}}{\Pr(T_i < T_j, T_i \leq l+t|T_i > l, T_j > l)}$$

$$= \frac{E_{ij}\left\{1\{\hat{S}_l(l+t|X_i(l),Z_i) < \hat{S}_l(l+t|X_j(l),Z_i)\}\int_l^{l+t} S(u|X_j(l),Z_i,u>l)\,S(du|X_i(l),Z,u>l)|T_i > l, T_j > l\right\}}{E_{ij}\left\{\int_l^{l+t} S(u|X_j(l),Z,u>l)\,S(u|X_i(l),Z,u>l)\right\}}$$

We assume that the event and censoring time are independent conditional on the variables, i.e. $C_i \coprod T_i|X_i(l), T_i > l, C_i > l$, and that the probability of being uncensored at the prediction horizon $l+t$ is bounded away from 0. This gives rise to the IPCW estimator

$$\hat{C}_l(t) = \frac{\sum_{i=1}^{n_l}\sum_{j=1}^{n_l} 1\{\hat{S}_l(l+t|X_i(l),Z) < \hat{S}_l(l+t|X_j(l),Z)\}1\{\tilde{T}_i < \tilde{T}_j\}1\{\tilde{T}_i \leq l+t, \Delta_\mathrm{i} = 1\}\widehat{W}_{ij}^{-1}}{\sum_{i=1}^{n_l}\sum_{j=1}^{n_l} 1\{\tilde{T}_i < \tilde{T}_j\}1\{\tilde{T}_i \leq l+t, \Delta_\mathrm{i} = 1\}\widehat{W}_{ij}^{-1}}$$

of $C_l(t)$, where $\widehat{W}_{ij} = \widehat{\Pr}(C_j > \tilde{T}_i|X_j(l), Z, \tilde{T}_j > l)\,\widehat{\Pr}(C_i \geq \tilde{T}_i|X_i(l), Z, \tilde{T}_i > l)$ is a weight, where the censoring probabilities used in the weight are obtained from a model to be specified (see below).

The C-index $C_l(t)$ is conditional on survival to age l and a separate estimated C-index is obtained for any combination of l and t ($l = 18, \dots, 50; t = 2,5,10$). We also considered an overall C-index which is combined across landmark ages. Consider the stacked landmark data set and let L_i denote the landmark age for record (row) i. Some individuals appear in more than one row in the stacked landmark data set and we define $ID(i)$ to be the unique identifier (ID number) for the individual in row i. The overall C-index is

$$C_{\mathrm{overall}}(t) = E_{ij}\left\{1(ID(i) \neq ID(j))1\left\{\hat{S}_{L_i}(L_i+t|X_i(l),Z) < \hat{S}_{L_j}(L_j+t|X_j(l),Z)\right\}|(T_i - L_i) < (T_j - L_i), (T_i - L_i) \leq t\right\}$$

where the expectation is with respect to two rows i, j in the stacked landmark data set. Inclusion of the indicator $1(ID(i) \neq ID(j))$ ensures that an individual is not compared with herself/himself. An estimator incorporating censoring weights is

$$\hat{C}_{\mathrm{overall}}(t) = \frac{\sum_{i=1}^{N}\sum_{j=1}^{N} 1\left\{\hat{S}_{L_i}(L_i+t|X_i(l),Z) < \hat{S}_{L_j}(L_j+t|X_j(l),Z)\right\}1\{\widetilde{(T_i} - L_i) < (\tilde{T}_j - L_i)\}1\{\tilde{T}_i - L_i \leq t, \Delta_\mathrm{i} = 1\}\widehat{W}_{ij}^{*-1}}{\sum_{i=1}^{N}\sum_{j=1}^{N} 1\{(\tilde{T}_i - L_i) < (\tilde{T}_j - L_j\}1\{(\tilde{T}_i - L_i) \leq t, \Delta_\mathrm{i} = 1\}\widehat{W}_{ij}^{*-1}}$$

where N is the total number of individuals in the stacked landmark data set and the weights are $\widehat{W}_{ij}^{*} = \widehat{\Pr}((C_j - L_j) > (\tilde{T}_i - L_i)|X_j(L_j), \tilde{T}_j > L_j)\,\widehat{\Pr}((C_i - L_i) \geq (\tilde{T}_i - L_i)|X_i(L_i), \tilde{T}_i > L_i)$.

We assumed that the probabilities in the weights $\widehat{W}_{ij}$ do not depend on $X_j(l)$ or Z and therefore used $\widehat{\Pr}(C_j > \tilde{T}_i|\tilde{T}_j > l)$ in place of s $\widehat{\Pr}(C_j > \tilde{T}_i|X_j(l), \tilde{T}_j > l)$ and $\widehat{\Pr}(C_i \geq \tilde{T}_i|\tilde{T}_i > l)$ in place of $\widehat{\Pr}$ $(C_i \geq \tilde{T}_i|X_i(l), Z, \tilde{T}_i > l)$. The probabilities were estimated separately from each landmark age using Kaplan-Meier estimates. A similar approach was used for the weights $\widehat{W}_{ij}^{*}$.

In summary we obtained $\hat{C}_{\mathrm{overall}}(t)$ for $t = 2,5,10$ and $\hat{C}_l(t)$ for $t = 2,5,10$ and $l = 18, \dots, 50$.

Brier score

The Brier score is the mean squared prediction error. As for the C-index, we obtained separate Brier scores at each landmark age and an overall brier score. In the absence of censoring an estimator of the Brier score is

$$\hat{B}_l(t) = \frac{1}{n_l}\sum_{i\in D_l}\{\hat{S}_{il}(l+t|X_i(l),Z_i) - I_i(T_i > l+t|T_i > l)\}^2$$

where $\hat{S}_{il}(l+t|X_i(l),Z_i)$ is the model-based estimated probability of survival to age $l+t$ for individual i in the landmark data set at age l, $I_i(t > l+t|T_i > l)$ is the observed indicator of survival to age $l+t$, and the sum is over the n_l individuals in landmark data set l (D_l). An estimator incorporating inverse probability of censoring weights is

$$\hat{B}_l(t) = \frac{1}{n_l}\sum_{i\in D_l} I(d_i = 1 \cup T_i > l+t)\{\hat{S}_{il}(l+t|X_i(l),Z_i) - I_i\left(T_i > l+t\middle|\tilde{T}_i > l\right)\}^2 \widehat{W}_i^{-1}$$

where d_i is the event indicator, $I(d_i = 1 \cup T_i > l+t)$ is an indicator taking value 1 for individuals who have the event or whose censoring age is after $t+l$, and zero otherwise, and $\widehat{W}_i = \widehat{\Pr}(C_i > \min(T_i^-, l+t)|\tilde{T}_i > l)$ is the probability of being censored beyond age $\min(T_i^-, l+t)$. The inverse probability of censoring weights were obtained using Kaplan-Meier estimates stratified by landmark age.

The overall Brier score estimator is

$$\hat{B}_{\text{overall}}(t) = \frac{1}{N}\sum_{i} I(d_i = 1 \cup T_i > L_i + t)\{\hat{S}_{iL_i}(L_i+t|X_i(L_i),Z_i) - I_i\left(T_i > L_i+t\middle|\tilde{T}_i > l\right)\}^2 \widehat{W}_i^{-1}$$

where the sum is over all rows in the stacked landmark data set and $\widehat{W}_i = \widehat{\Pr}(C_i > \min(T_i^-, L_i + t)|\tilde{T}_i > L_i)$.

Brier scores were also obtained under a null model using Kaplan-Meier estimates of the survival probabilities stratified by landmark age but with no other predictors. These are denoted $\hat{B}_{l,\text{null}}(t)$ and $\hat{B}_{\text{overall,null}}(t)$. The percentage reduction in the Brier score from a given model compared with the null model was calculated using $100(\hat{B}_{l,\text{null}}(t) - \hat{B}_l(t))/\hat{B}_{l,\text{null}}(t)$ and $100(\hat{B}_{\text{overall,null}}(t) - \hat{B}_{\text{overall}}(t))/\hat{B}_{\text{overall,null}}(t)$.

In summary we obtained $\hat{B}_{\text{overall}}(t)$ for $t = 2,5,10$ and $\hat{B}_l(t)$ for $t = 2,5,10$ and $l = 18,\dots,50$, and the corresponding percentages reductions in the Brier score relative to the null model.

Calibration plots

After selecting the final model, calibration plots were obtained to show graphically the agreement between predicted survival probabilities from the model and the 'true' probabilities. The steps for creating these plots were as follows:

Steps (i)-(iii) are the same as described earlier, in the Overview section of eAppendix 3.
(iv) The predicted 2-year survival probabilities from landmark age l were divided into quintiles and we obtained the mean predicted 2-year survival probability for individuals within each quintile, denoted $\bar{S}(2)_{l,Q1}, \bar{S}(2)_{l,Q2}, \bar{S}(2)_{l,Q3}, \bar{S}(2)_{l,Q4}, \bar{S}(2)_{l,Q5}$. We also obtained the Kaplan-Meier estimate of 2-year survival for the individuals within each quintile, denoted $KM(2)_{l,Q1}, KM(2)_{l,Q2}, KM(2)_{l,Q3}, KM(2)_{l,Q4}, KM(2)_{l,Q5}$. The same was done for 5-year and 10-year survival.
(v) Steps (i)-(iv) were repeated 200 times and for each $l = 18,\dots,50$ we obtained the average of each $\bar{S}(2)_{l,Q1},\dots,\bar{S}(2)_{l,Q5}$ and the average of each $KM(2)_{l,Q1}, K\dots, KM(2)_{l,Q5}$ across the 200 samples..
(vi) The averaged $\bar{S}(2)_{l,Q1},\dots,\bar{S}(2)_{l,Q5}$ from step (v) were plotted against the averaged $KM(2)_{l,Q1}, K\dots, KM(2)_{l,Q5}$.

Calibration plots for landmark ages 20, 30, 40 and 50 are shown in eFigure 4. In a well-calibrated model the five points lie on the $y = x$ line.

eAppendix 4. Software

All analyses were performed using R. The landmark models described in eAppendix 2 can be fitted easily using the coxph function from the survival package after some rearrangement of the data.[13] Some of the data rearrangement can be performed using the dynpred package,[14] for example using the cutLM function, though we did not use that here. Estimated survival probabilities can be obtained using 'predict' after coxph, though special code was written to obtain the predicted survival probabilities from Model 4, which included time-varying coefficients.

There exist various packages for obtaining C-indexes and Brier scores. None of the existing functions for estimating the C-index appear to accommodate a stratified baseline hazard, and so we used bespoke code. We used 'pew' from the dynpred package to estimate the Brier scores; this requires pre-estimation of matrices of predicted survival and censoring probabilities.

The multivariate mixed model used to obtain the additional predictors $X^*(l)$ for Model 6 was fitted using the lme function from the nlme package.[15] Existing software, including the nlme package, does not appear to allow out-of-sample predictions from mixed models. We therefore used bespoke code which is available from https://github.com/ruthkeogh/landmark_CF .

eAppendix 5. Final model specification

R code for obtaining estimated survival probabilities from the final model is provided at https://github.com/ruthkeogh/landmark_CF. This includes csv files containing estimated cumulative baseline hazards for each landmark age ($l = 18, \ldots, 50$).

eAppendix 6. Comparisons with other models

In an analysis of the French CF Registry Nkam et al reported a cross-validated C-statistic of 0.90 for prediction of 3-year survival.[16] They did not report a Brier score. Aside from focusing on 3-year survival and using different set of predictors, there are a number of differences between their approach and ours. They used a composite outcome of death and transplant, and for their logistic regression analysis, they excluded individuals who were censored before the end of the 3-year follow-up period.

Liou et al used a logistic regression analysis of the US CF Registry to predict 5-year survival.[17] A calibration plot showed good performance using a validation data set. However, they did not present measures of predictive performance that are comparable to those in this paper. Mayer-Hamblett et al also used a logistic regression analysis of the US CF Registry to develop a model for predicting 2-year survival.[18] They presented an ROC curve but did not report an area under the ROC curve, which could be compared to our C-Index. They presented sensitivities and specificities, and positive- and negative predictive values, finding that their model was better at predicting who would survive 2 years than who would die.

McCarthy et al developed the CF-ABLE score using logistic regression modelling of data from the CF population in Ireland.[19] Based on a validation data set, the area under the ROC curve was 0.82 for 4-year survival, though it is not clear how censoring was treated.

eFigure 1. Summary of data exclusions and creation of data set for analysis.

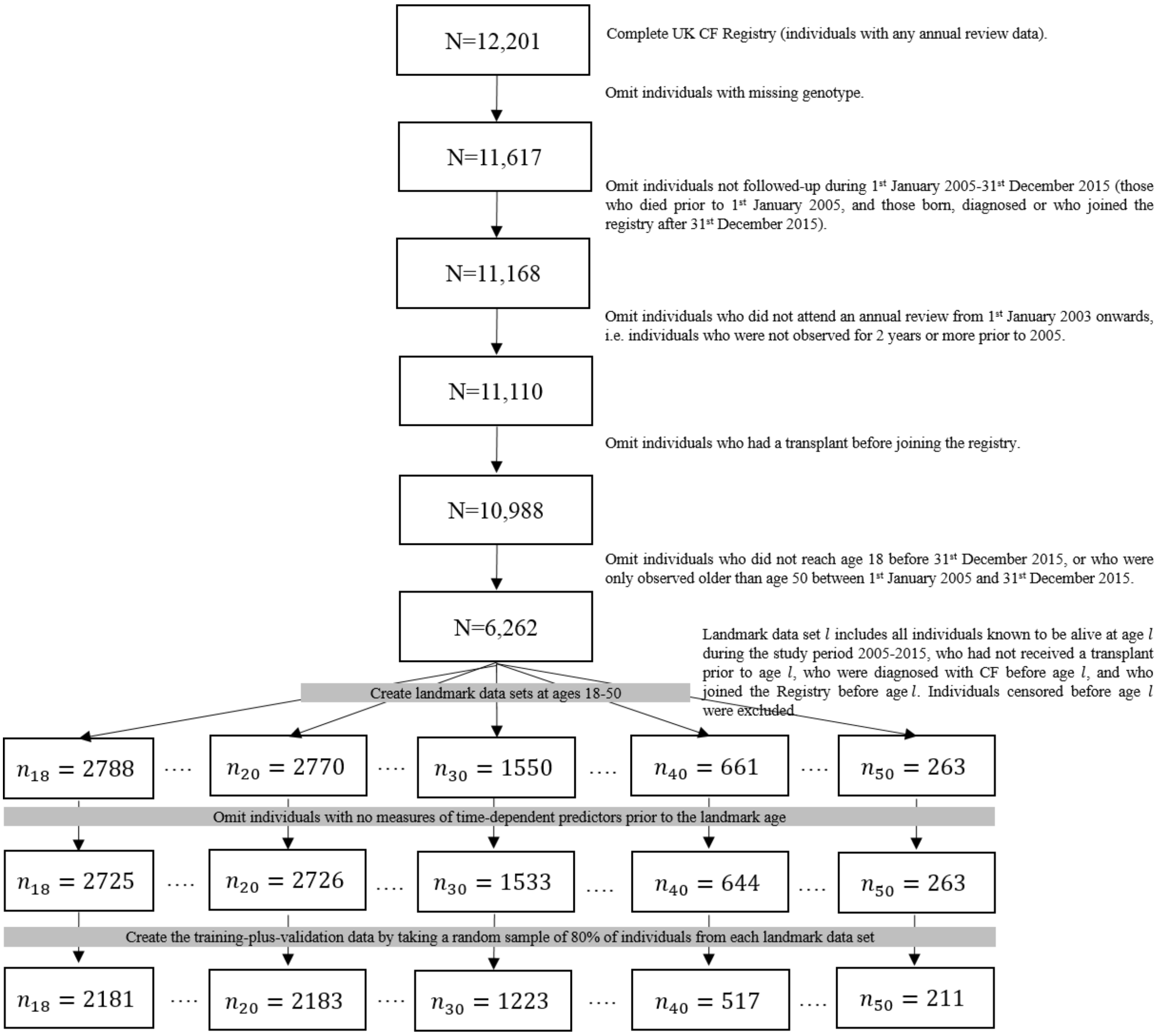

eTable 1. Summary of number of individuals, deaths, censorings and total person time at risk in each landmark data set. The stacked data set is formed by combining the landmark data sets.

Landmark age	No. of individuals	Number of deaths: N (%) Within 2 years	Within 5 years	Within 10 years	Number of censorings: N (%) Within 2 years	Within 5 years	Within 10 years
18	2725	63 (2.3)	171 (6.3)	255 (9.4)	500 (18.3)	1243 (45.6)	2290 (84.0)
19	2756	86 (3.1)	206 (7.5)	290 (10.5)	522 (18.9)	1244 (45.1)	2294 (83.2)
20	2726	104 (3.8)	218 (8.0)	303 (11.1)	505 (18.5)	1215 (44.6)	2239 (82.1)
21	2622	96 (3.7)	209 (8.0)	291 (11.1)	497 (19.0)	1221 (46.6)	2185 (83.3)
22	2526	107 (4.2)	206 (8.2)	273 (10.8)	477 (18.9)	1194 (47.3)	2104 (83.3)
23	2431	99 (4.1)	196 (8.1)	258 (10.6)	463 (19.0)	1159 (47.7)	2022 (83.2)
24	2326	85 (3.7)	182 (7.8)	234 (10.1)	501 (21.5)	1136 (48.8)	1970 (84.7)
25	2225	80 (3.6)	167 (7.5)	219 (9.8)	486 (21.8)	1088 (48.9)	1878 (84.4)
26	2079	82 (3.9)	160 (7.7)	216 (10.4)	439 (21.1)	1026 (49.4)	1760 (84.7)
27	1953	81 (4.1)	153 (7.8)	205 (10.5)	412 (21.1)	960 (49.2)	1647 (84.3)
28	1801	74 (4.1)	145 (8.1)	189 (10.5)	386 (21.4)	909 (50.5)	1540 (85.5)
29	1675	59 (3.5)	117 (7.0)	164 (9.8)	385 (23.0)	882 (52.7)	1436 (85.7)
30	1533	61 (4.0)	112 (7.3)	149 (9.7)	355 (23.2)	822 (53.6)	1323 (86.3)
31	1396	52 (3.7)	102 (7.3)	135 (9.7)	330 (23.6)	772 (55.3)	1205 (86.3)
32	1286	49 (3.8)	110 (8.6)	132 (10.3)	338 (26.3)	721 (56.1)	1112 (86.5)
33	1185	44 (3.7)	99 (8.4)	124 (10.5)	316 (26.7)	671 (56.6)	1011 (85.3)
34	1062	46 (4.3)	92 (8.7)	114 (10.7)	283 (26.6)	588 (55.4)	899 (84.7)
35	981	45 (4.6)	84 (8.6)	104 (10.6)	253 (25.8)	533 (54.3)	807 (82.3)
36	881	43 (4.9)	74 (8.4)	94 (10.7)	228 (25.9)	473 (53.7)	750 (85.1)
37	796	32 (4.0)	60 (7.5)	83 (10.4)	200 (25.1)	425 (53.4)	685 (86.1)
38	732	31 (4.2)	56 (7.7)	74 (10.1)	181 (24.7)	373 (51.0)	623 (85.1)
39	688	27 (3.9)	56 (8.1)	72 (10.5)	163 (23.7)	346 (50.3)	581 (84.4)
40	644	19 (3.0)	47 (7.3)	68 (10.6)	141 (21.9)	319 (49.5)	544 (84.5)
41	618	20 (3.2)	48 (7.8)	71 (11.5)	124 (20.1)	327 (52.9)	518 (83.8)
42	606	30 (5.0)	50 (8.3)	72 (11.9)	131 (21.6)	314 (51.8)	501 (82.7)
43	579	24 (4.1)	57 (9.8)	72 (12.4)	130 (22.5)	302 (52.2)	485 (83.8)
44	530	19 (3.6)	45 (8.5)	64 (12.1)	141 (26.6)	277 (52.3)	447 (84.3)
45	497	20 (4.0)	47 (9.5)	65 (13.1)	131 (26.4)	274 (55.1)	415 (83.5)
46	425	23 (5.4)	42 (9.9)	57 (13.4)	96 (22.6)	229 (53.9)	353 (83.1)
47	391	23 (5.9)	42 (10.7)	54 (13.8)	93 (23.8)	215 (55.0)	327 (83.6)
48	347	14 (4.0)	35 (10.1)	45 (13.0)	98 (28.2)	202 (58.2)	292 (84.1)
49	307	15 (4.9)	34 (11.1)	39 (12.7)	92 (30.0)	184 (59.9)	260 (84.7)
50	263	17 (6.5)	31 (11.8)	37 (14.1)	71 (27.0)	154 (58.6)	218 (82.9)

eTable 2. Summary of number of measurements of FEV1%, FVC% and weight used in multivariate mixed models fitted up to each landmark age. Results shown are the median, interquartile range (IQR) and range of the number of measurements of each variable up to age l for individuals in the lth landmark data set ($l = 18, \ldots, 50$).

	FEV1%			FVC%			Weight		
Landmark age	Median	IQR	Range	Median	IQR	Range	Median	IQR	Range
18	7	(5,10)	(1,20)	7	(5,10)	(1,20)	8	(5,11)	(1,21)
19	7	(5,10)	(1,21)	7	(5,10)	(1,21)	8	(5,11)	(1,22)
20	7	(5,10)	(1,22)	7	(5,10)	(1,22)	8	(5,11)	(1,22)
21	7	(5,10)	(1,21)	7	(5,10)	(1,21)	8	(5,11)	(1,21)
22	8	(5,10)	(1,21)	8	(5,10)	(1,21)	8	(5,11)	(1,21)
23	8	(5,10)	(1,22)	8	(5,10)	(1,22)	8	(5,11)	(1,22)
24	8	(5,11)	(1,24)	8	(5,11)	(1,24)	8	(6,11)	(1,24)
25	8	(6,11)	(1,24)	8	(5,11)	(1,24)	8	(6,11)	(1,24)
26	8	(5,11)	(1,24)	8	(5,11)	(1,24)	8	(6,11)	(1,25)
27	8	(5,11)	(1,23)	8	(5,11)	(1,23)	8	(6,11)	(1,22)
28	8	(6,11)	(1,23)	8	(6,11)	(1,23)	8	(6,11)	(1,23)
29	8	(5,11)	(1,22)	8	(5,11)	(1,22)	8	(6,11)	(1,22)
30	8	(6,11)	(1,20)	8	(6,11)	(1,20)	9	(6,11)	(1,21)
31	9	(6,11)	(1,21)	9	(6,11)	(1,21)	9	(6,12)	(1,21)
32	9	(6,11.75)	(1,23)	9	(6,11)	(1,23)	9	(6,12)	(1,23)
33	8	(5,11)	(1,21)	8	(5,11)	(1,21)	9	(5,12)	(1,22)
34	8	(5,11)	(1,23)	8	(5,11)	(1,23)	8	(5,12)	(1,24)
35	8	(5,11)	(1,19)	8	(5,11)	(1,19)	8	(5,12)	(1,19)
36	8	(5,11)	(1,19)	8	(5,11)	(1,19)	8	(5,12)	(1,19)
37	8	(5,11)	(1,19)	8	(5,11)	(1,19)	8	(5,11)	(1,18)
38	8	(5,11)	(1,19)	8	(5,11)	(1,18)	8	(5,11)	(1,18)
39	7	(4,11)	(1,18)	7	(4,11)	(1,18)	8	(4,11)	(1,19)
40	7	(4,10)	(1,19)	7	(4,10)	(1,19)	7	(4.5,11)	(1,18)
41	7	(4,10)	(1,18)	7	(4,10)	(1,18)	7	(5,11)	(1,18)
42	7	(4,10)	(1,17)	7	(4,10)	(1,17)	7	(4,10)	(1,17)
43	7	(4,10)	(1,18)	7	(4,10)	(1,18)	7	(4,10)	(1,18)
44	7	(5,10)	(1,19)	7	(5,10)	(1,19)	7	(5,11)	(1,19)
45	7	(5,10)	(1,20)	7	(5,10)	(1,20)	7	(5,10)	(1,20)
46	7	(5,10)	(1,18)	7	(5,10)	(1,18)	7	(5,10)	(1,18)
47	7	(5,10)	(1,19)	7	(5,10)	(1,19)	8	(5,10)	(1,18)
48	7	(5,10)	(1,20)	7	(5,10)	(1,20)	8	(5,10.5)	(1,20)
49	8	(5,11)	(1,21)	8	(5,11)	(1,21)	8	(5,11)	(1,20)
50	8	(5,11)	(1,16)	8	(5,11)	(1,16)	8	(5,11)	(1,16)

FEV1%: Percent predicted forced expiratory volume in 1 second.
FVC%: Percent predicted forced vital capacity.

eTable 3. Descriptive statistics at landmark ages 20, 30, 40, and 50. Summaries are given as number (N) and percent for categorical variables and as median and interquartile range (IQR) for continuous variables.

Variable		Landmark age 20		Landmark age 30		Landmark age 40		Landmark age 50	
		N	%	N	%	N	%	N	%
Sex	Male	1443	52.9	863	56.3	385	59.8	160	60.8
	Female	1283	47.1	670	43.7	259	40.2	103	39.2
Genotype	2 copies	1549	56.8	820	53.5	263	40.8	87	33.1
	1 copy	956	35.1	567	37.0	312	48.4	141	53.6
	Other	221	8.1	146	9.5	69	10.7	35	13.3
P. aeruginosa	No	1127	41.3	471	30.7	234	36.3	107	40.7
	Yes	1599	58.7	1062	69.3	410	63.7	156	59.3
B. cepacia	No	2621	96.1	1445	94.3	604	93.8	253	96.2
	Yes	105	3.9	88	5.7	40	6.2	10	3.8
S. aureus	No	1580	58.0	940	61.3	410	63.7	167	63.5
	Yes	1146	42.0	593	38.7	234	36.3	96	36.5
MRSA	No	2651	97.2	1480	96.5	628	97.5	255	97.0
	Yes	75	2.8	53	3.5	16	2.5	8	3.0
Pancreatic insufficiency	No	224	8.2	189	12.3	150	23.3	87	33.1
	Yes	2502	91.8	1344	87.7	494	76.7	176	66.9
CF related diabetes	No	1968	72.2	914	59.6	382	59.3	158	60.1
	Yes	758	27.8	619	40.4	262	40.7	105	39.9
Hospitalisation (not for IVs)	No	2649	97.2	1483	96.7	626	97.2	250	95.1
	Yes	77	2.8	50	3.3	18	2.8	13	4.9
Number of hospital IV days	0 days	1648	60.5	958	62.5	458	71.1	187	71.1
	1-14 days	487	17.9	274	17.9	109	16.9	37	14.1
	15-28 days	245	9.0	125	8.2	36	5.6	19	7.2
	29+ days	346	12.7	176	11.5	41	6.4	20	7.6
Number of home IV days	0 days	1852	67.9	931	60.7	425	66.0	188	71.5
	1-14 days	340	12.5	227	14.8	85	13.2	28	10.6
	15-28 days	229	8.4	132	8.6	50	7.8	20	7.6
	29+ days	305	11.2	243	15.9	84	13.0	27	10.3
		Median	IQR	Median	IQR	Median	IQR	Median	IQR
Age of diagnosis (years)		0.3	(0.1, 2.0)	0.7	(0.1, 3.5)	2.0	(0.3, 18.1)	13.0	(1.0, 36.0)
Calendar year		2010	(2008, 2013)	2011	(2009, 2013)	2011	(2008, 2013)	2012	(2009, 2014)
FEV1%		69.4	(52.1, 85.6)	60.5	(42.8, 78.6)	55.3	(38.1, 74.7)	53.9	(36.6, 72.3)
FVC%		83.0	(68.6, 95.8)	79.9	(63.4, 92.3)	77.6	(61.2, 91.3)	74.7	(62.6, 89.6)
Weight (kg)		57.0	(50.4, 65.3)	63.0	(55.3, 72.2)	66.1	(58.9, 75.6)	69.0	(60.5, 79.5)
Height (cm)		166.3	(160.0, 173.1)	169.0	(162.0, 176.0)	169.0	(162.9, 175.0)	169.5	(162.0, 176.0)

FEV1%: Percent predicted forced expiratory volume in 1 second.
FVC%: Percent predicted forced vital capacity.
IV: Intravenous antibiotic therapy.
MRSA: Methicillin-resistant *Staphylococcus aureus*

eTable 4. Results from the holdout data. Comparison between predicted survival probabilities from the final model and numbers of survivors and deaths within 2, 5 and 10 years from landmark ages 20, 30, 40 and 50. For 2-, 5-, and 10-year survival we excluded those who were censored before 2, 5 and 10 years of follow-up respectively. Note that due to small numbers in some predicted probability groups we do not expect the observed percentages surviving to exactly match the predicted survival probabilities.

		2-year survival		5-year survival		10-year survival	
Landmark age	Probability of 2-year, 5-year or 10-year survival from final model	No. (%) who survived 2 years	No. (%) who died within 2 years	No. (%). who survived 5 years	No. (%) who died within 5 years	No. (%) who survived 10 years	No. (%) who died within 10 years
20	[0,0.7]	4 (57%)	3 (43%)	13 (48%)	14 (52%)	8 (20%)	32 (80%)
	(0.7,0.9]	43 (90%)	5 (10%)	51 (78%)	14 (22%)	13 (50%)	13 (50%)
	(0.9,0.95]	43 (93%)	3 (7%)	50 (94%)	3 (6%)	8 (73%)	3 (27%)
	(0.95,1]	341 (99%)	3 (1%)	166 (98%)	3 (2%)	15 (83%)	3 (17%)
30	[0,0.7]	2 (50%)	2 (50%)	5 (25%)	15 (75%)	3 (10%)	26 (90%)
	(0.7,0.9]	22 (73%)	8 (27%)	27 (73%)	10 (27%)	5 (28%)	13 (72%)
	(0.9,0.95]	37 (95%)	2 (5%)	21 (84%)	4 (16%)	4 (57%)	3 (43%)
	(0.95,1]	160 (99%)	2 (1%)	63 (97%)	2 (3%)	3 (75%)	1 (25%)
40	[0,0.7]	0 (0%)	1 (100%)	0 (0%)	5 (100%)	2 (14%)	12 (86%)
	(0.7,0.9]	5 (71%)	2 (29%)	17 (74%)	6 (26%)	1 (50%)	1 (50%)
	(0.9,0.95]	11 (100%)	0 (0%)	10 (100%)	0 (0%)	1 (100%)	0 (0%)
	(0.95,1]	81 (99%)	1 (1%)	31 (100%)	0 (0%)	0	0
50	[0,0.7]	3 (100%)	0 (0%)	1 (25%)	3 (75%)	0 (0%)	5 (100%)
	(0.7,0.9]	5 (71%)	2 (29%)	4 (67%)	2 (33%)	0 (0%)	2 (100%)
	(0.9,0.95]	8 (89%)	1 (11%)	5 (100%)	0 (0%)	1 (100%)	0 (0%)
	(0.95,1]	20 (100%)	0 (0%)	4 (100%)	0 (0%)	1 (100%)	0 (0%)
	[0,0.7]						

eTable 5. Overall C-Indexes and Brier scores for prediction of 2-year, 5-year and 10-year survival from a model including FEV1% as the only predictor and from a model including two treatment variables in addition to the 16 predictors included in the final model (Model 2 in Table 3 of the main text).

	Results from the final model (Model 2: Table 3 of the main text)		Model using FEV1% predicted as the only predictor[a]		Additionally including two treatment variables in Model 2[b]	
	C-Index	Brier score	C-Index	Brier score	C-Index	Brier score
2-year survival	0.873	0.036	0.842	0.038	0.876	0.035
5-year survival	0.843	0.076	0.813	0.081	0.844	0.075
10-year survival	0.804	0.133	0.775	0.141	0.805	0.133

a We repeated the final model with FEV1% predicted as the only predictor. Other features of the model were as in Model 2.
b We assessed the impact on predictive performance of including two treatments that were included in the model of Nkam et al for the French Registry: use of oxygen therapy and use of non-invasive ventilation.[16] Nkam et al also investigated use of oral corticosteroids, but there was insufficient data on use of this treatment in the UK data. We created binary variables at each landmark age, which indicate whether an individual had ever used each treatment in the past. The adjusted hazard ratio associated with oxygen use was 1.75 (95% CI 1.50-2.05) and the adjusted hazard ratio associated with non-invasive ventilation is 1.15 (95% CI 0.92-1.43). Therefore both oxygen therapy and non-invasive ventilation are associated with an increased mortality hazard (though the association for non-invasive ventilation is not statistically significant), because these treatments are used by sicker patients. The estimates do not have a causal interpretation.

eFigure 2. Comparison of landmark-age-specific C-indexes for 2-year, 5-year and 10-year survival from Model 1 (separate models from each landmark age) and Model 2 (supermodel).

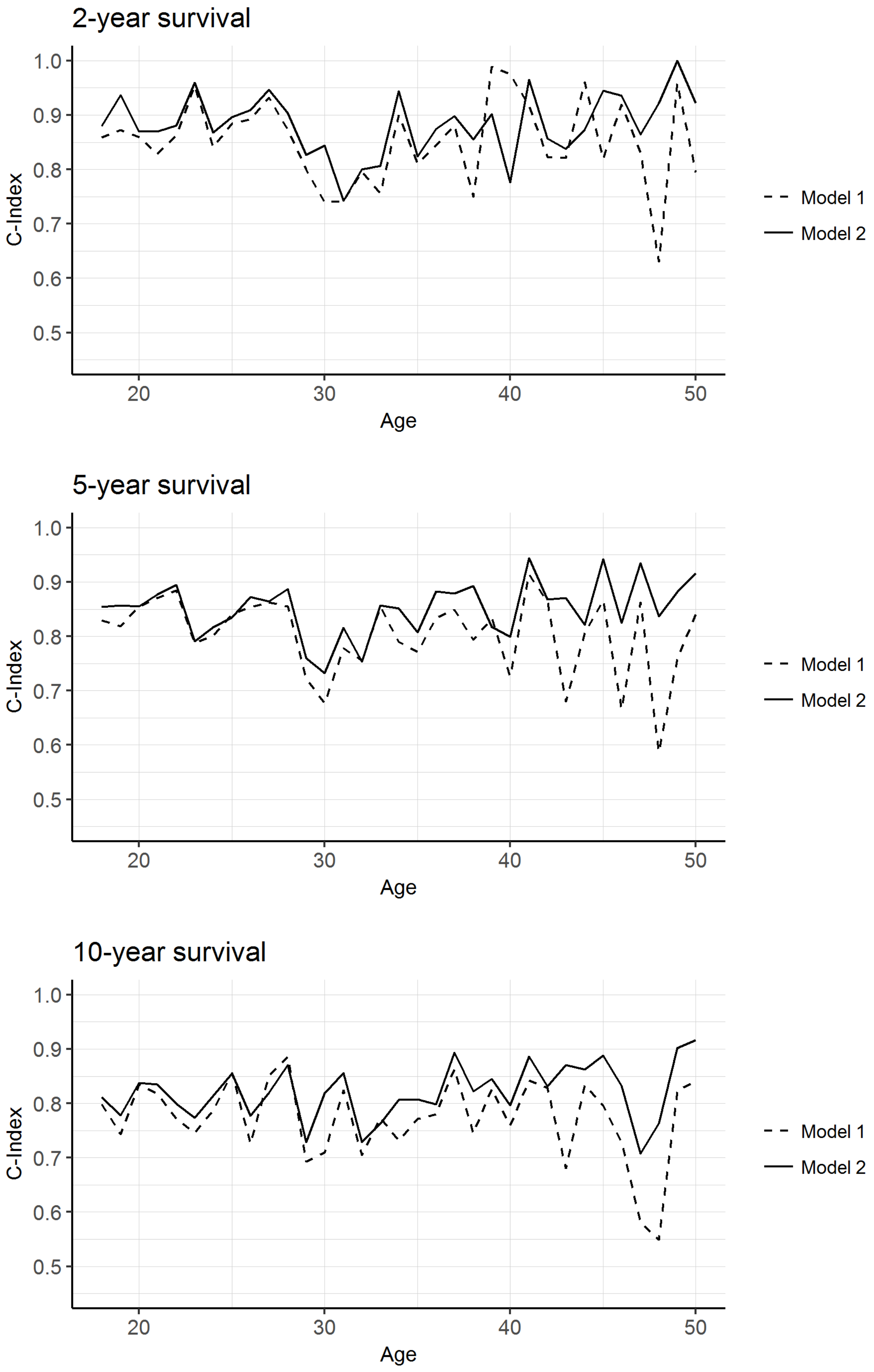

eFigure 3. Comparison of landmark-age-specific Brier scores for 2-year, 5-year and 10-year survival from Model 1 (separate models from each landmark age) and Model 2 (supermodel).

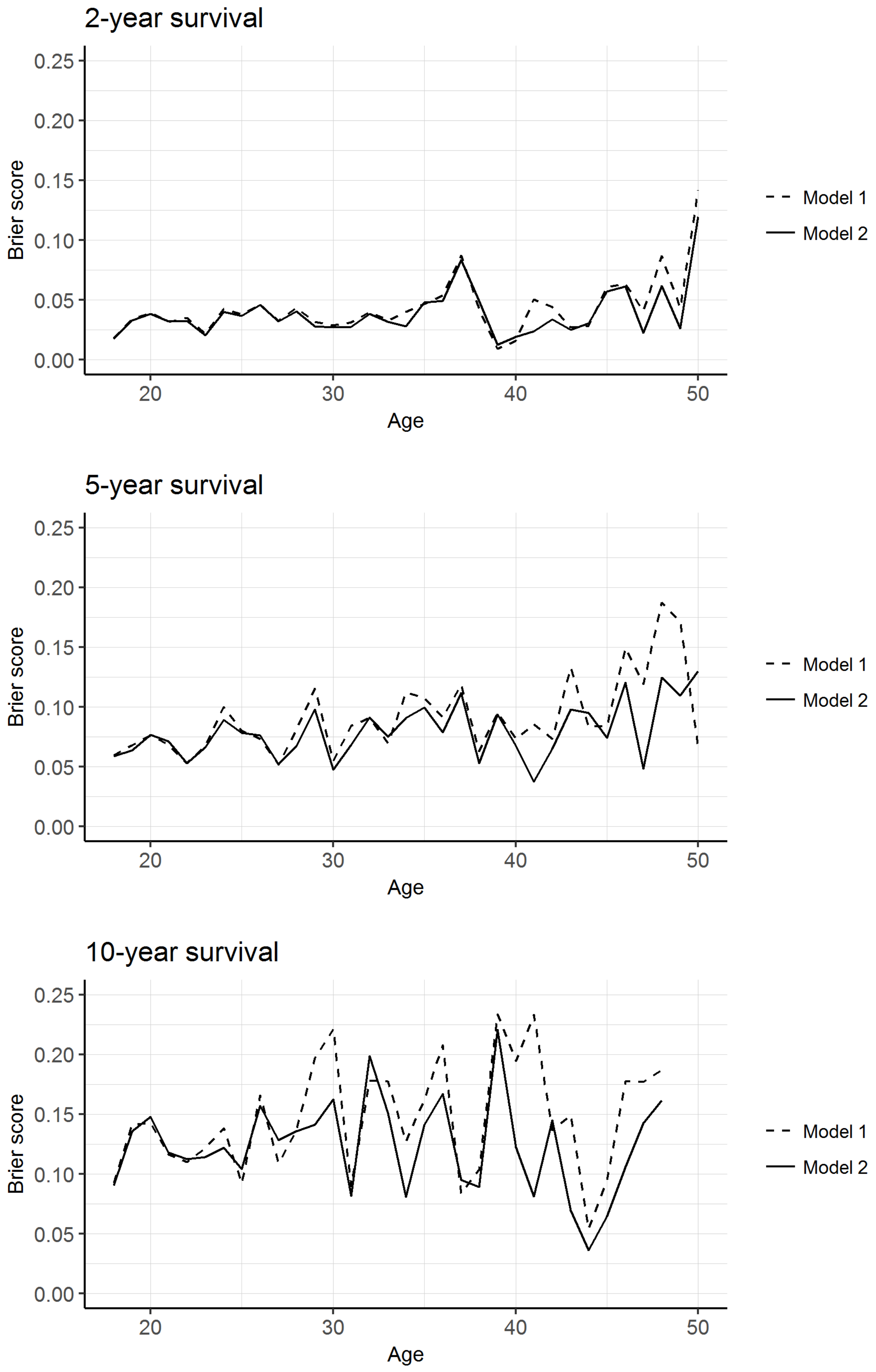

eFigure 4. Calibration plots using the final model (Model 2) for prediction of 2-year, 5-year and 10-year survival from landmark ages 20, 30, 40 and 50. The vertical axis shows the mean model-based x-year survival probability (x=2, 5,10) in quintiles of the model-based probabilities. The horizontal axis shows the mean x-year survival probability obtained using Kaplan-Meier estimates in quintiles of the model-based probabilities. The five points have been joined by a line.

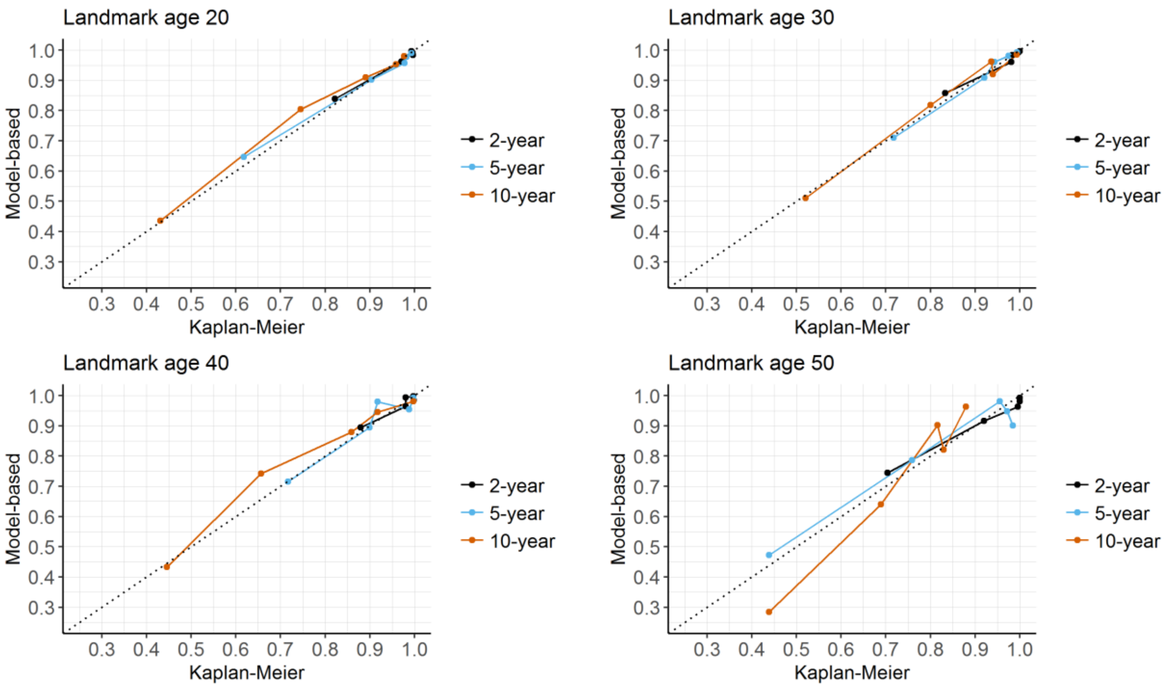

eFigure 5. Predicted survival curves from landmark age 20 for example individuals in groups defined by 5-year survival probabilities. For individuals in the Registry at age 20 between 2013 and 2015 we obtained their predicted 5-year survival probabilities and categorized into groups with 5-year survival probabilities <0.5, (0.5,0.7,], (0.7,0.8], (0.8,0.9], (0.9,0.95], (0.95,0.99], (0.99, 1]. An example individual was created for each group.

(i) Characteristics of example individuals[a] in groups defined by 5-year survival probability.

5-year survival probability group	<0.5	(0.5,0.7]	(0.7,0.8]	(0.8,0.9]	(0.9,0.95]	(0.95,0.99]	(0.99,1]
Example person	1	2	3	4	5	6	7
**Males, Females[b]**							
Genotype (no. copies of F508del)	1, 2	2	2	2	2	2	1
Age of diagnosis (years)	0	0	0	0	0	0	1, 3
FEV1%	22, 26	28, 35	43, 39	44, 51	59, 61	78, 70	97, 98
FVC%	32, 39	48. 53	58, 54	64, 72	75, 77	90, 90	103, 106
Weight (kg)	48, 46	53, 47	51, 49	56, 48	57, 53	65, 56	73, 64
Height (cm)	167, 159	169, 156	167, 160	174, 158	170, 158	173, 161	177, 164
P. aeruginosa	Yes	Yes	Yes	Yes	Yes	Yes	No
B. cepacia	No	No	No	No	No	No	No
S. aureus	No	Yes, No	Yes, No	No	Yes, No	No	No
MRSA	No	No	No	No	No	No	No
Pancreatic insufficiency	Yes	Yes	Yes	Yes	Yes	Yes	Yes
CF related diabetes	Yes	Yes	Yes	No, Yes	No	No	No
Hospitalisation (not for IVs)	No	No	No	No	No	No	No
Number of hospital IV days	29+	15-28, 29+	29+, 1-14	1-14, 15-28	0	0	0
Number of hospital IV days	0	1-14, 29+	0	0, 1-14	0	0	0

[a]We created an example individual for each group using the median values of the continuous predictors and the most common value of each categorical variable within that group. For hospital and home IV days we obtained the median number of days and then assigned the relevant category. This was done separately for males and females.
[b] Values are shown as 'male, female', except were the value for males and females was the same.
FEV1%: Percent predicted forced expiratory volume in 1 second.
FVC%: Percent predicted forced vital capacity.
IV: Intravenous antibiotic therapy.
MRSA: Methicillin-resistant *Staphylococcus aureus*.

(ii) Predicted survivor curves based on the final model for example individuals with characteristics shown in the table above.

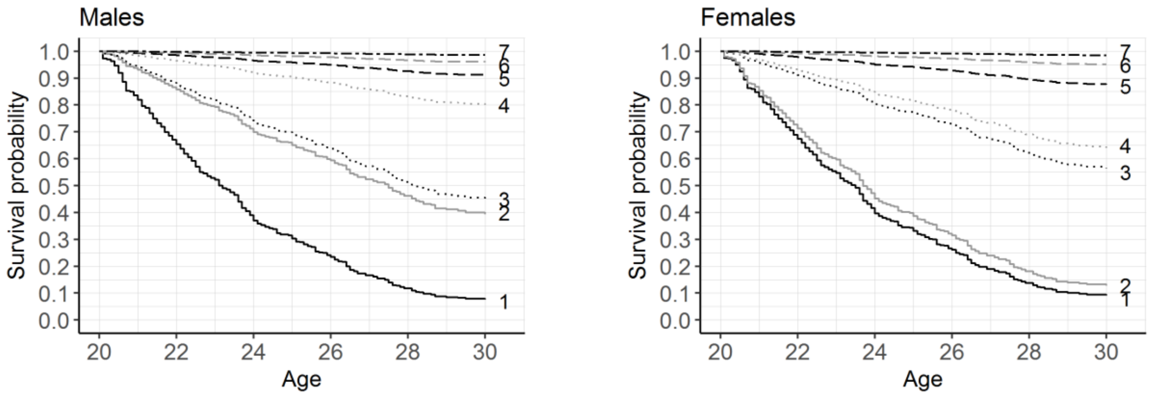

eFigure 6. Predicted survival curves from landmark age 30 for example individuals in groups defined by 5-year survival probabilities. For individuals in the Registry at age 30 between 2013 and 2015 we obtained their predicted 5-year survival probabilities and categorized into groups with 5-year survival probabilities <0.5, (0.5,0.7], (0.7,0.8], (0.8,0.9], (0.9,0.95], (0.95,0.99], (0.99, 1]. An example individual was created for each group.

(i) Characteristics of example individuals[a] in groups defined by 5-year survival probability.

5-year survival probability group	<0.5	(0.5,0.7]	(0.7,0.8]	(0.8,0.9]	(0.9,0.95]	(0.95,0.99]	(0.99,1]
Example person	1	2	3	4	5	6	7
**Males/Females[b]**							
Genotype (no. copies of F508del)	2	2	2	2	2	2	1
Age of diagnosis (years)	0, 0	0, 0	0, 1	0, 1	0, 0	0, 1	5, 3
FEV1%	29, 25	24, 38	35, 32	36, 43	51, 54	71, 76	91, 97
FVC%	31, 36	49, 57	57, 50	63, 61	69, 70	88, 89	100, 102
Weight (kg)	48, 48	64, 47	60, 52	62, 55	69, 56	70, 58	77, 68
Height (cm)	170, 156	172, 156	173, 156	172, 162	173, 163	174, 162	178, 166
P. aeruginosa	No, Yes	Yes	Yes	Yes	Yes	Yes	Yes
B. cepacia	Yes, No	No	No	No	No	No	No
S. aureus	No	No	No	No	No	No	No, Yes
MRSA	No	No	No	No	No	No	No
Pancreatic insufficiency	Yes	Yes	Yes	Yes	Yes	Yes	Yes
CF related diabetes	Yes	Yes	Yes	Yes	Yes	No	No
Hospitalization (not for IVs)	No	No	No	No	No	No	No
Number of hospital IV days	29+	29+	29+, 0	0	0	0	0
Number of hospital IV days	0, 29+	0, 29+	0, 29+	0	0	0	0

[a]We created an example individual for each group using the median values of the continuous predictors and the most common value of each categorical variable within that group. This was done separately for males and females.
[b] Values are shown as 'male, female', except were the value for males and females was the same.
FEV1%: Percent predicted forced expiratory volume in 1 second.
FVC%: Percent predicted forced vital capacity.
IV: Intravenous antibiotic therapy.
MRSA: Methicillin-resistant *Staphylococcus aureus*.

(ii) Predicted survivor curves based on the final model for example individuals with characteristics shown in the table above.

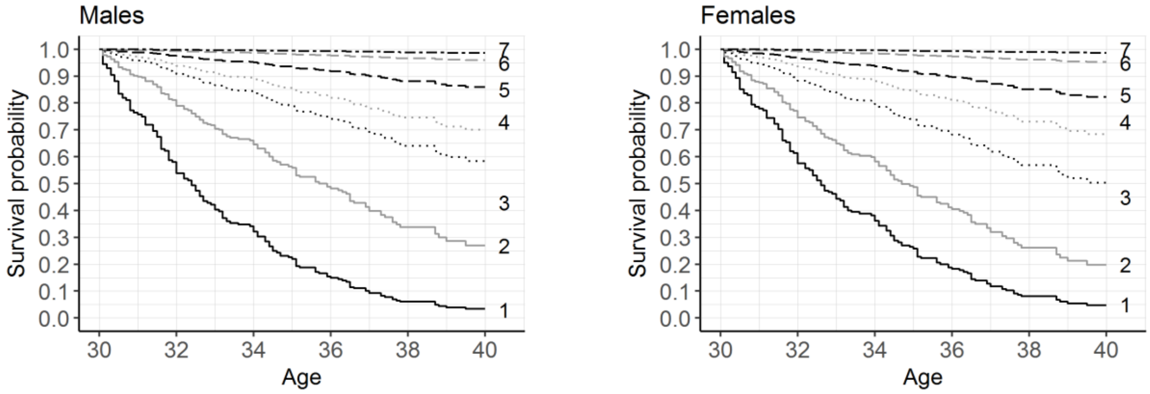

eFigure 7. Predicted survival curves from landmark age 40 for example individuals in groups defined by 5-year survival probabilities. For individuals in the Registry at age 40 between 2013 and 2015 we obtained their predicted 5-year survival probabilities and categorized into groups with 5-year survival probabilities <0.5, (0.5,0.7], (0.7,0.8], (0.8,0.9], (0.9,0.95], (0.95,0.99], (0.99, 1]. An example individual was created for each group.

(i) Characteristics of example individuals[a] in groups defined by 5-year survival probability. Results are not shown for groups of less than 5 individuals.

5-year survival probability group	<0.5	(0.5,0.7]	(0.7,0.8]	(0.8,0.9]	(0.9,0.95]	(0.95,0.99]	(0.99,1]
Example person	1	2	3	4	5	6	7
**Males, Females[b]**							
Genotype (no. copies of F508del)	-	2	2	2,1	2,1	1	1
Age of diagnosis (years)	-	2, 0	1, 0	1, 4	3, 3	2, 14	29, 13
FEV1%	-	27, 25	31, 28	38, 41	51, 47	68, 65	92, 92
FVC%	-	42, 43	60, 45	64, 59	70, 66	93, 81	97, 96
Weight (kg)	-	67	64	63	68	75	85
Height (cm)	-	173	170	173	176	176	175
P. aeruginosa	-	Yes	Yes	Yes	Yes	Yes	No
B. cepacia	-	No	No	No	No	No	No
S. aureus	-	No	No	No	No	No	No, Yes
MRSA	-	No	No	No	No	No	No
Pancreatic insufficiency	-	Yes	Yes	Yes	Yes	Yes	No
CF related diabetes	-	Yes	Yes	Yes	No, Yes	No	No
Hospitalisation (not for IVs)	-	No	No	No	No	No	No
Number of hospital IV days	-	29+	15-28, 1-14	1-14	0, 1-14	0	0
Number of hospital IV days	-	29+, 1-14	0, 1-14	0	0	0	0

[a]We created an example individual for each group using the median values of the continuous predictors and the most common value of each categorical variable within that group. For hospital and home IV days we obtained the median number of days and then assigned the relevant category. This was done separately for males and females.
[b] Values are shown as ‘male, female’, except were the value for males and females was the same.
FEV1%: Percent predicted forced expiratory volume in 1 second.
FVC%: Percent predicted forced vital capacity.
IV: Intravenous antibiotic therapy.
MRSA: Methicillin-resistant *Staphylococcus aureus*.

(ii) Predicted survivor curves based on the final model for example individuals with characteristics shown in the table above.

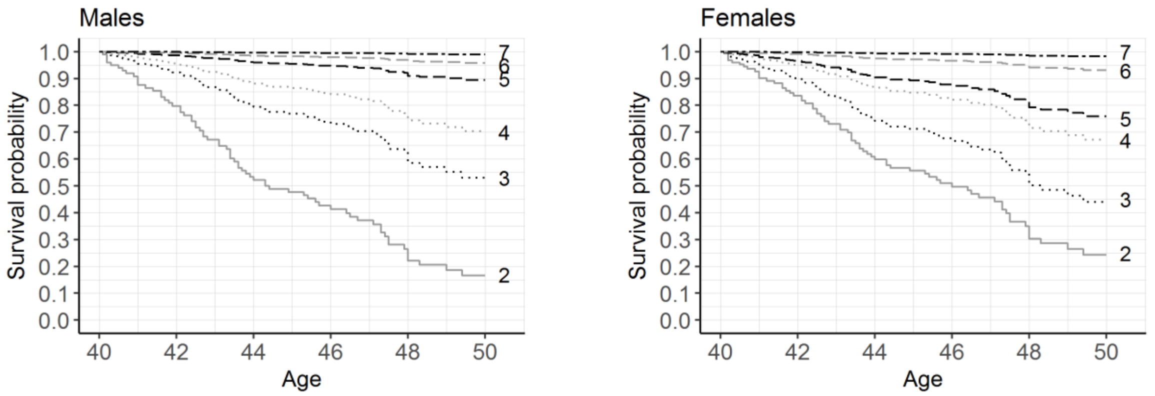

eFigure 8. Predicted survival curves from landmark age 50 for example individuals in groups defined by 5-year survival probabilities. For individuals in the Registry at age 50 between 2013 and 2015 we obtained their predicted 5-year survival probabilities and categorized into groups with 5-year survival probabilities <0.5, (0.5,0.7], (0.7,0.8], (0.8,0.9], (0.9,0.95], (0.95,0.99], (0.99, 1]. An example individual was created for each group.

(i) Characteristics of example individuals[a] in groups defined by 5-year survival probability. Results are not shown for groups of less than 5 individuals.

5-year survival probability group	<0.5	(0.5,0.7]	(0.7,0.8]	(0.8,0.9]	(0.9,0.95]	(0.95,0.99]	(0.99,1]
Example person	1	2	3	4	5	6	7
**Males/Females[b]**							
Genotype (no. copies of F508del)	-	1, 2	2, -	2, 1	2, 1	1	1, -
Age of diagnosis (years)	-	4, 1	4, -	1	6, 28	28, 34	39, -
FEV1%	-	31, 30	27, -	48, 49	55, 64	82, 76	113, -
FVC%	-	51, 64	63, -	72, 69	76, 81	91, 90	108, -
Weight (kg)	-	65, 55	76, -	76, 61	80, 66	79, 65	86, -
Height (cm)	-	172, 158	174, -	17, 165	176, 162	176, 163	177, -
P. aeruginosa	-	Yes	Yes, -	Yes	No, Yes	No	No, -
B. cepacia	-	No	No, -	No	No	No	No, -
S. aureus	-	No	No, -	No	No	No	No, -
MRSA	-	No	No, -	No	No	No	No, -
Pancreatic insufficiency	-	Yes	Yes, -	Yes	Yes	Yes, No	No, -
CF related diabetes	-	Yes	Yes, -	Yes, No	No	No	No, -
Hospitalisation (not for IVs)	-	No	No, -	No	No	No	No, -
Number of hospital IV days	-	1-14	1-14, -	0	0	0	0, -
Number of hospital IV days	-	0	1-14, -	0	1-14	0	0, -

[a]We created an example individual for each group using the median values of the continuous predictors and the most common value of each categorical variable within that group. For hospital and home IV days we obtained the median number of days and then assigned the relevant category. This was done separately for males and females.

[b] Values are shown as 'male, female', except were the value for males and females was the same.

FEV1%: Percent predicted forced expiratory volume in 1 second.

FVC%: Percent predicted forced vital capacity.

IV: Intravenous antibiotic therapy.

MRSA: Methicillin-resistant *Staphylococcus aureus*.

(ii) Predicted survivor curves based on the final model for example individuals with characteristics shown in the table above.

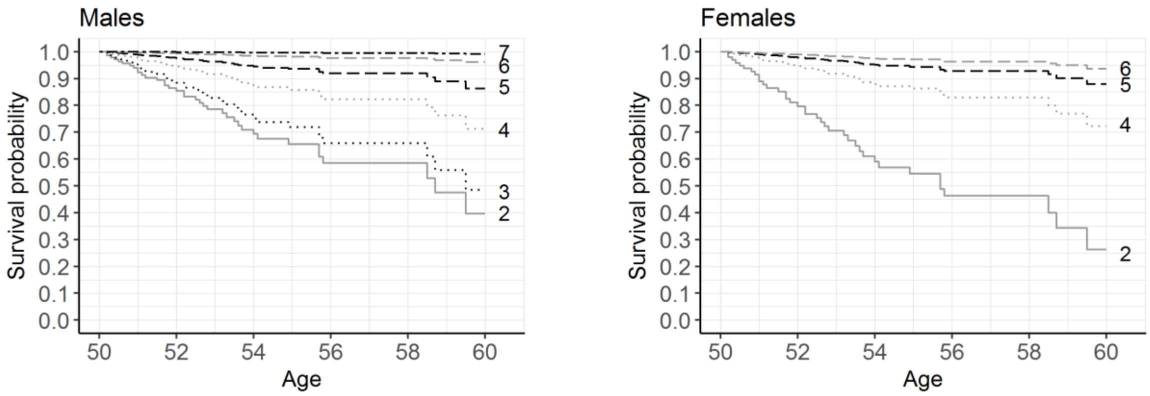

eFigure 9. [This is a colour version of Figure 2 in the main text.] Plots showing the distribution of 2-, 5- and 10-year survival probabilities from landmark ages 20, 30, 40 and 50 for individuals in the Registry at those ages between 2013 and 2015.

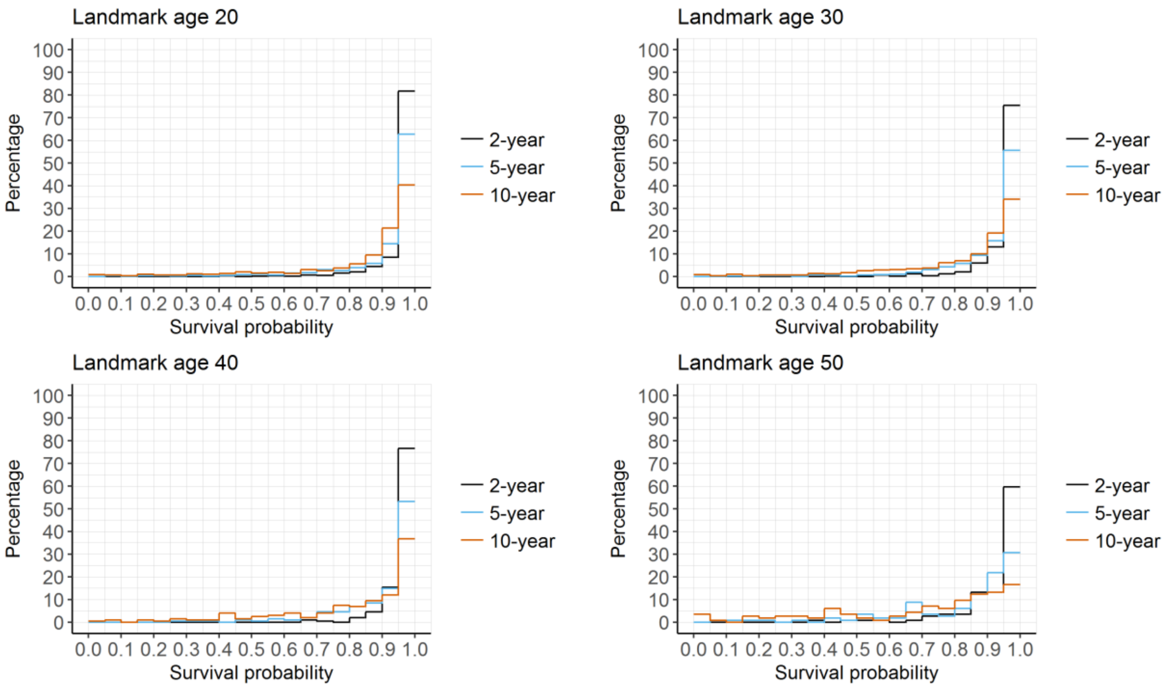

References

1. Cox, D. R. Models and Life-Tables Regression. *J. R. Stat. Soc. Ser. B.* **34,** 187–220 (1972).

2. Cox, D. R.. Partial likelihood. *Biometrika* **62,** 269–276 (1975).

3. Breslow, N. Discussion of the paper by D. R. Cox. *J. R. Stat. Soc. Ser. B* **34,** 216–217 (1972).

4. Yong, F., Cai, T., Wei, L. & Tian, L. Classical model selection. in *Handbook of Survival Analysis* (eds. Klein, J., van Houwelingen, H., Ibrahim, J. & Scheike, T.) (CRC Press, 2014).

5. Pencina, M. J., D'Agostino, R. B., D'Agostino, R. B. & Vasan, R. S. Evaluating the added predictive ability of a new marker: From area under the ROC curve to reclassification and beyond. *Stat. Med.* **27,** 157–172 (2008).

6. Uno, H., Cai, T., Pencina, M. J., D'Agostino, R. B. & Wei, L. J. On the C-statistics for evaluating overall adequacy of risk prediction procedures with censored survival data. *Stat. Med.* **30,** 1105–1117 (2011).

7. Gerds, T. A., Kattan, M. W., Schumacher, M. & Yu, C. Estimating a time-dependent concordance index for survival prediction models with covariate dependent censoring. *Stat. Med.* **32,** 2173–2184 (2013).

8. Antolini, L., Boracchi, P. & Biganzoli, E. A time-dependent discrimination index for survival data. *Stat. Med.* **24,** 3927–3944 (2005).

9. Graf, E., Schmoor, C., Sauerbrei, W. & Schumacher, M. Assessment and comparison of prognostic classification schemes for survival data. *Stat. Med.* **18,** 2529–2545 (1999).

10. Gerds, T. A. & Schumacher, M. Consistent Estimation of the Expected Brier Score in General Survival Models with Right-Censored Event Times. *Biometrical J.* **48,** 1029–1040 (2006).

11. van Houwelingen, H. C. & Putter, H. *Dynamic prediction in clinical survival analysis*. (CRC Press/Chapman and Hall, 2012).

12. Kuhn, M. & Johnson, K. Over-Fitting and Model Tuning. In *Applied Predictive Modelling.* pp. 61–92 (Springer, 2013).

13. Therneau, T. A Package for Survival Analysis in S. R package version 2.38. https://CRAN.R-project.org/package=survival (2015).

14. Putter, H. Dynpred package. https://cran.r-project.org/package=dynpred (2015).

15. Pinheiro, J., Bates, D., DebRoy, S., Sarkar, D. & R Core Team. nlme: Linear and Nonlinear Mixed Effects Models. R package version 3.1-131.1. https://CRAN.R-project.org/package=nlme (2018).

16. Nkam, L., Lambert, J., Latouche, A., Bellis, G., Burgel, P. R. & Hocine, M. N. A 3-year prognostic score for adults with cystic fibrosis. *J. Cyst. Fibros.* **16,** 701-708 (2017).

17. Liou, T. G., Adler, F.R., FitzSimmons, S. C., Cahill, B. C., Hibbs, J. R. & Marshall, B. C. Predictive 5-year survivorship model of cystic fibrosis. *Am. J. Epidemiol.* **153,** 345–352 (2001).

18. Mayer-Hamblett, N., Rosenfeld, M., Emerson, J., Goss, C. H. & Aitken, M. L. Developing Cystic Fibrosis Lung Transplant Referral Criteria Using Predictors of 2-Year Mortality. *Am. J. Respir. Crit. Care Med.* **166,** 1550–1555 (2002).

19. McCarthy, C., Dimitrov, B. D., Meurling, I. J., Gunaratnam, C. & McElvaney, N. G. The CF-ABLE score: A novel clinical prediction rule for prognosis in patients with cystic fibrosis. *Chest* **143,** 1358–1364 (2013).
